# Spontaneous Patterning of Binary Ligand Mixtures on CdSe Nanocrystals: From Random to Janus Packing

**DOI:** 10.1021/acsnano.2c12676

**Published:** 2023-03-09

**Authors:** Orian Elimelech, Meirav Oded, Daniel Harries, Uri Banin

**Affiliations:** † The Institute of Chemistry and The Center for Nanoscience and Nanotechnology, The Hebrew University of Jerusalem, Jerusalem 9190401, Israel; ‡ The Fritz Haber Center, The Hebrew University of Jerusalem, Jerusalem 9190401, Israel

**Keywords:** CdSe nanocrystals, ligand exchange, ligand patterning, isothermal titration calorimetry, interligand interaction

## Abstract

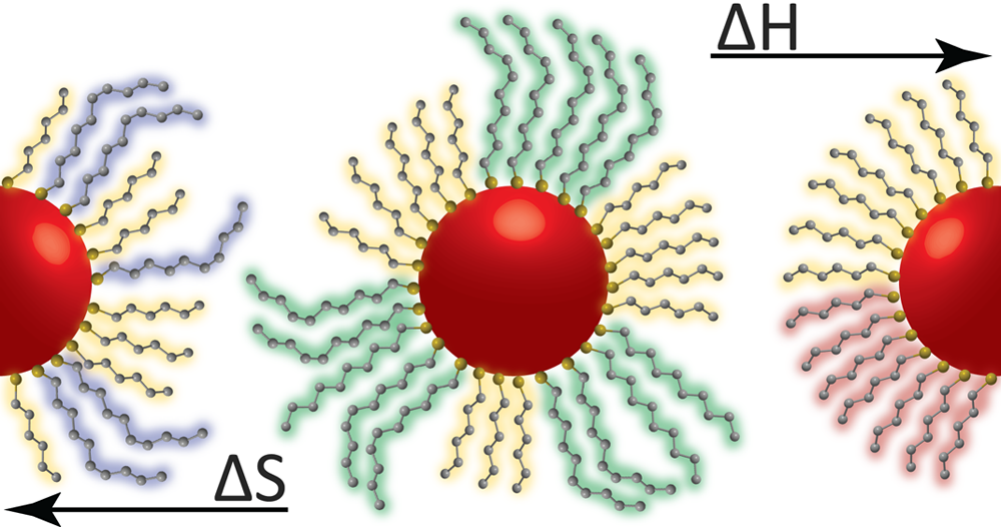

Binary compositions of surface ligands are known to improve the colloidal stability and fluorescence quantum yield of nanocrystals (NCs), due to ligand–ligand interactions and surface organization. Herein, we follow the thermodynamics of a ligand exchange reaction of CdSe NCs with alkylthiol mixtures. The effects of ligand polarity and length difference on ligand packing were investigated using isothermal titration calorimetry (ITC). The thermodynamic signature of the formation of mixed ligand shells was observed. Correlating the experimental results with thermodynamic mixing models has allowed us to calculate the interchain interactions and to infer the final ligand shell configuration. Our findings demonstrate that, in contrast to macroscopic surfaces, the small dimensions of the NCs and the subsequent increased interfacial region between dissimilar ligands allow the formation of a myriad of clustering patterns, controlled by the interligand interactions. This work provides a fundamental understanding of the parameters determining the ligand shell structure and should help guide smart surface design toward NC-based applications.

Surface ligands not only impact the chemical compatibility of nanocrystals (NCs)^1,2^ but also provide passivation of surface dangling bonds,^3−5^ thereby enabling control over the optical and electronic properties of the NCs.^6−8^ Multicomponent ligand coatings of NCs were demonstrated to improve their colloidal stability^9^ and their fluorescence quantum yield.^10,11^ Beyond its influence on the NCs’ properties, the ability to engineer multiligand shell patterns provides important control over NC self-assembly toward complex structures,^12^ as well as their incorporation in many applications.^13^

The study of ligand patterning on inorganic NCs has mostly concentrated on Au nanoparticles (NPs),^14−17^ and only a few of these combined theory and experiment to resolve the interplay between interchain interactions and the resulting shell organization.^18,19^ Surface ligand organization is dictated by the balance of configurational entropy and the contribution of ligand–ligand interactions to the enthalpy and entropy of mixing.^15,18^ By mixing chemically distinct ligands, it is possible to tune their surface organization, because full phase separation is typically enthalpically driven, while mixing between ligands is entropically favored.

Chemical distinction between mixed ligands can be introduced by altering chain polarity,^14−17,19^ bulkiness,^20^ and length.^9,18^ For example, mixing short and long ligands liberates conformational and rotational degrees of freedom of the longer ligands unimpeded by the shorter chains at the interface.^9,18^ This enthalpy–entropy balance leads to the final ligand patterning on the NC, which can show Janus packing, random mixing, or intermediate ligand clustering.^18^ Unlike macroscopic systems, where phase separation is distinctly observed between regions of different composition, for microscopically small systems, the demixing transition is less sharp, mostly due to the relative increased contribution of the fraction of interfacial ligands residing in the region between ligand clusters. The ligand organization and the thermodynamics of these finite macroscopic systems are, therefore, more sensitive to details of the ligand–ligand interactions.^19^

To elucidate the interchain enthalpy–entropy balance at nanometric dimensions, we studied two main systems of NCs with a binary composition of capping ligands. First, we followed the effect of the ligands’ length difference (Δ*l*) on the interchain interactions and, as a result, on the distribution of the ligands within that shell. In the second system, we examined the effect of differences in degree of polarity between the ligands composing the shell, while maintaining similar ligand lengths.

In experiments, the binary shell was obtained via a ligand exchange reaction of oleate (−O_2_CR)-coated CdSe NCs with a mixture of linear alkylthiols (R′SH and R″SH) at different ratios (Scheme 1):
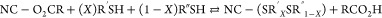
1

**Scheme 1:**
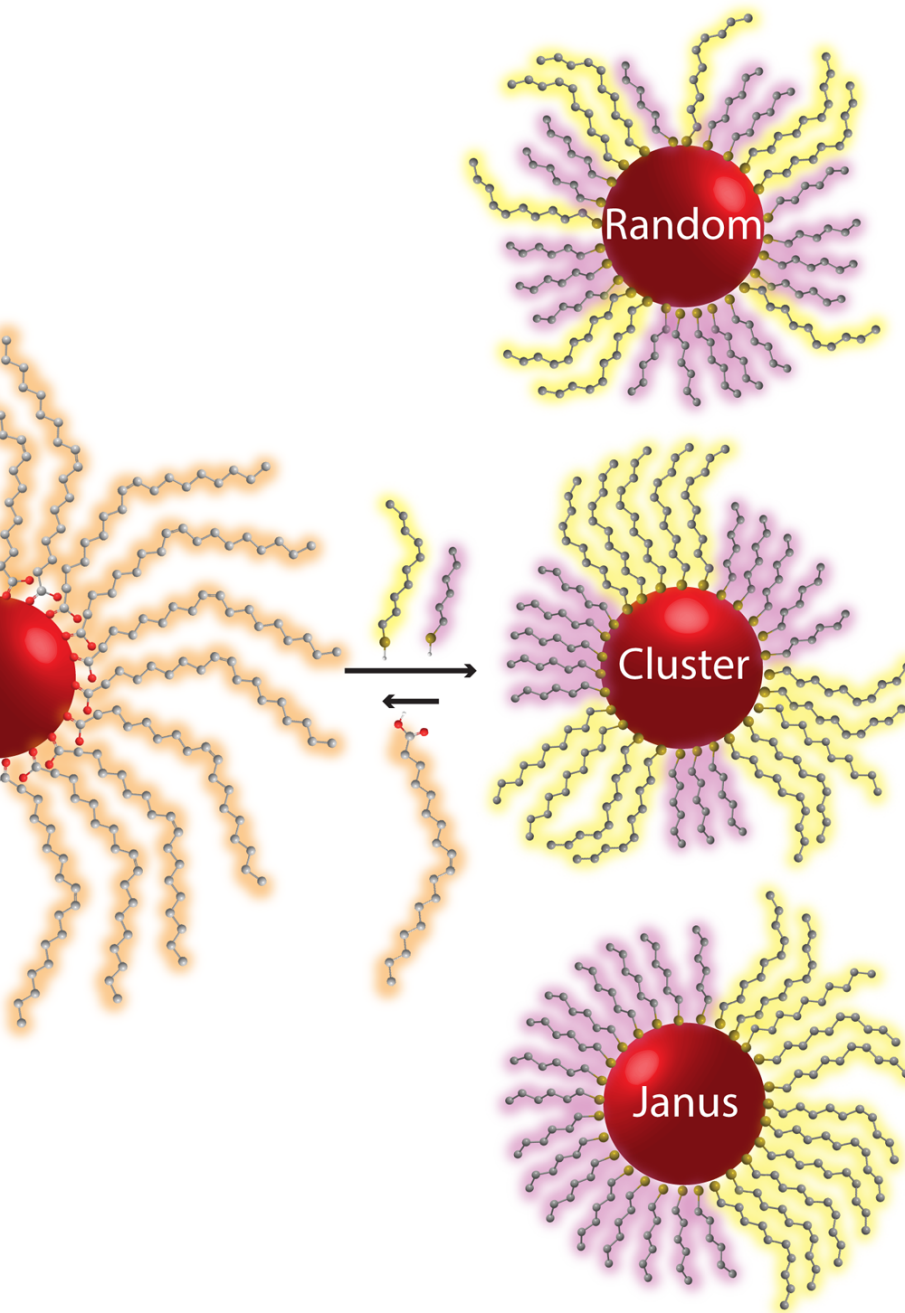
Investigated ligand exchange reaction between oleate-coated CdSe NCs and a binary mixture of thiolated ligands. Shown are the possible organizations of the thiolated ligand shells, classified into random packing (involving full mixing of the two ligands), clustering (where segregated patches of the different ligands form), and Janus behavior (where the two ligand types occupy separate regions on the NC surface).

As was reported for similar systems, the exchange reaction involves proton migration from the alkylthiol molecules to the oleate, thereby facilitating the latter’s detachment from the Cd^2+^ ion as oleic acid and the attachment of the alkylthiolate, in accordance with the *x*-type ligand exchange mechanism.^21−24^

In the first system, designed to study the effect of variations in ligand length, Δ*l*, several sets of binary shells were compared, each set consisting of 1-hexanethiol (C6SH), as the reference point, at varying ratios with respect to the longer ligands (i.e., 1-decaenthiol, C10SH; 1-tetradecanethiol, C14SH; 1-octadecanethiol, C18SH). Additionally, similar length differences were compared for different ligand lengths (i.e., C6SH:C10SH vs C14SH:C18SH). In the second system, the C6SH ligand was mixed with 1*H*,1*H*,2*H*,2*H*-perfluoro-1-hexanethiol (C6SH(F)). The thermodynamic parameters of the exchange reaction were extracted by isothermal titration calorimetry (ITC) experiments and fitted to mixing models, allowing to resolve and dissect the different contributions to the thermodynamics of ligand shell patterning.

## Results and Discussion

**Figure 1:**
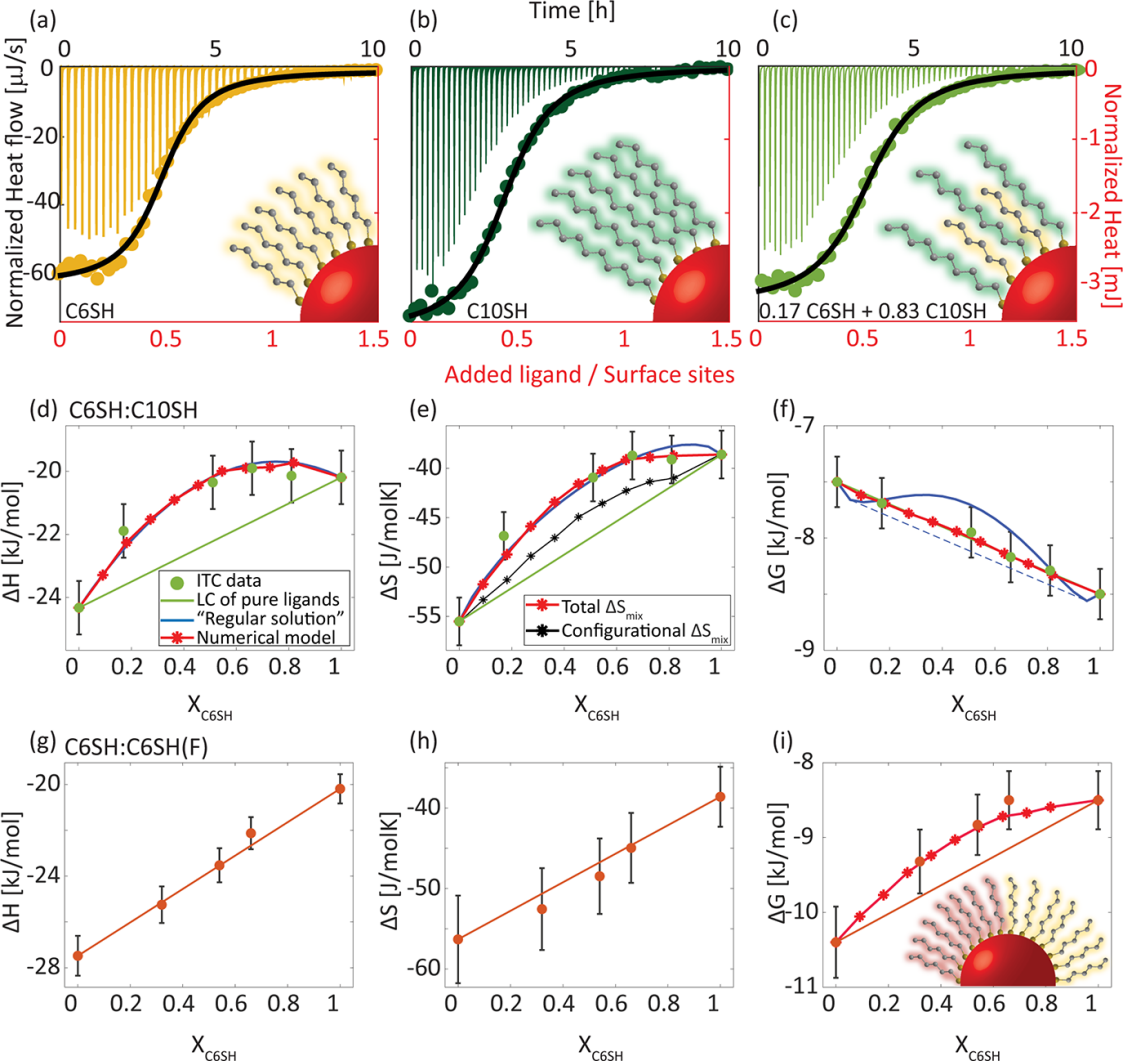
ITC results for ligand exchange reaction of oleate-coated CdSe NCs (*d* = 3.0 nm) with pure and mixed ligands. (a–c) Real-time ITC thermograms (black axis) and the corresponding titration curves and fittings (dots, black line, respectively, red axis) for the exchange with (a) pure hexanethiol (C6SH), (b) pure decanethiol (C10SH), and (c) a C6SH_0.17_C10SH_0.83_ binary composition. Inset: Illustration of the yielded systems. (d–f) The extracted thermodynamic parameters: (d) enthalpy, (e) entropy, and (f) Gibbs free energy, for the exchange with pure C6SH (X_C6SH_ = 1), C10SH (X_C6SH_ = 0), and their binary composition (0 < X_C6SH_ < 1) (green dots). Solid green line represents the calculated linear combination (LC) of the pure ligands. Solid blue line represents fitting to the regular solution model. Red asterisk and line represent the numerical model. Black asterisk and line represent the calculated configurational entropy. (g–i) The extracted thermodynamic parameters: (g) enthalpy, (h) entropy, and (i) Gibbs free energy, for the exchange reaction with pure C6SH, fluorinated hexanethiol (C6SH(F)), and their binary compositions (orange dots). Solid orange line represents the calculated linear combination of the pure ligands. Red asterisk and line represent the numerical model. Inset: Illustration of the surface ligand organization.

Figure 1 shows the ITC thermogram for the exchange reaction of oleate-coated CdSe NCs (*d* = 3 nm) at 303 K with pure C6SH (a), pure C10SH (c), and a binary composition of C6SH_0.17_C10SH_0.83_ (b). All thermograms were normalized to the concentrations of Cd^2+^ surface sites and the injected ligands. Integration of the thermogram peaks provides titration curves, representing the heat change as a function of the ratio between the added ligand and the Cd^2+^ surface sites (Figure 1a–c, overlay). All curves were fitted to a single-site exchange reaction model, presented in our previous work.^21^ This model considers both the detachment of the native oleate ligand and the binding of the alkylthiol, similar to the approaches recently presented in other models for exchange reactions on the NC surface,^25−29^ thus allowing to extract a single set of thermodynamic parameters, including the reaction enthalpy (Δ*H*), entropy (Δ*S*), and Gibbs free energy (Δ*G*) (SI, Section 4). Although the NC surface is expected to contain multiple types of surface sites,^30,31^ this heterogeneity is apparently masked in the binary shell, probably due to the expected sparser packing (as will be discussed in the following), which resulted in thermodynamically identical facet and edge surface sites.

All measured reactions are exothermic (Δ*H* < 0) and favorable (Δ*G* < 0), as is expected for the exchange of a carboxylate binding group with a thiol. As reported elsewhere, the reaction involves an entropy loss (Δ*S* < 0), mainly due to the replacement of the unorganized oleate ligand shell with the more organized alkylthiol ligand shell.^21^

As ligand chain length increases, the exchange with the pure ligand exhibits an increase in exothermicity (−20.2 and −24.3 kJ/mol for C6SH and C10SH, respectively) and entropy loss (−39 and −56 J/mol K for C6SH and C10SH, respectively), leading to a minor change in Δ*G* gain (−8.5 and −7.5 kJ/mol for C6SH and C10SH, respectively), in agreement with previous results.^21,22,27^ For the exchange reaction with a binary mixture of ligands, the thermodynamic parameters are intermediate between the values for the two pure constituent ligands (Δ*H* = −21.8 kJ/mol, Δ*S* = −47 J/mol K and Δ*G* = −7.7 kJ/mol). Closer inspection of these values reveals that although C10SH is the majority component, the ITC-extracted Δ*H* and Δ*S* values more closely resemble those for C6SH, pointing to some contribution of the mixed ligand shell formation to the thermodynamics of the reaction.

To further unravel the effect of ligand composition on the extent of mixing, we analyzed the exchange with additional C6SH:C10SH ratios. Figure 1d, e, and f (green circles) present the extracted Δ*H*, Δ*S*, and Δ*G* values of the measured compositions, respectively. Much like the exchange reaction with the pure ligands, the binary mixtures showed exothermic reaction heat and a loss of entropy. Moreover, as the molar fraction, XC6SH, of the C6SH increases, the exothermicity of the reaction decreases, along with the entropic loss, leading to overall only minor changes in Δ*G*. Similar enthalpy–entropy compensation (EEC) was previously observed in related NC systems (Figure S10).^21,22,32^

To resolve the contribution of ligand mixing, we compared the ITC-extracted results to a simple weighted linear combination (LC) of the pure ligands’ thermodynamic parameters (Figure 1d–f, green line). All calculations use the ligand ratio added to the solution. Due to the high and similar affinity (Δ*G*) of the pure alkylthiols to the NC surface, the final ligand ratio on the NC surface should be similar to the one in solution. Further justification for this assumption is presented in the SI, Section 5, by using a Langmuir model for competitive adsorption.^33^

As the linear combination considers no mutual influence between both ligands, it represents the results expected for a fully phase separated system on the NC surface, whereas any deviation from this line is necessarily associated with the thermodynamic contribution due to mixing. This comparison reveals compensating positive deviations in the enthalpy (lower exothermicity) and in the entropy (lower entropy loss), resulting in overall negligible deviations in Δ*G* from the linear combination line.

Despite the similar polarity of both ligands, the mixing between the different lengths is enthalpically disfavored, yet entropically favorable, which can be ascribed to the increase in the number of microstates in the mixed system along the contact interfacial regimes. These trends are more pronounced with increasing molar ratio of C10SH (decreasing XC6SH), suggesting higher perturbation of the C6SH ligands toward the C10SH ligand packing in the shell, than vice versa. From the perspective of a shell consisting of short ligands, embedded fractions of longer ligands should not significantly hamper interchain van der Waals (vdW) interactions. Indeed, the shorter ligand may even gain interactions with the longer ligand, due to possible folding-over of the longer ligands, which can lead to more compact packing, thus also lowering the entropy upon mixing. However, from the longer ligand’s perspective, fractions of surrounding shorter ligands will reduce ligand–ligand vdW interactions, due to their smaller number of methylene groups, resulting in lower exothermicity (Δ*H*_mix_ > 0). In addition, the presence of the shorter ligands provides additional rotational free volume to the longer chains, which directly affects their conformational entropy (Δ*S*_mix_ > 0), as was observed by others.^9,18^

Although the Δ*G* values we find are similar to the linear combination, suggesting no thermodynamic preference toward mixing (Δ*G*_mix_ ≈ 0), the observed deviations from linear combination, seen as lower exothermicity and entropy loss in the case of the C6SH:C10SH compositions, suggest some degree of mixing between both ligands. We note that while the solvent should contribute to the overall measured interactions in solution and on the NC surface, this contribution is already included in the calculated linear combination; thus the deviations in the parameters are attributed solely to the ligand–ligand interactions.

To further examine the interplay between mixing enthalpy and entropy, we studied a binary mixture of C6SH and C6SH(F). The largely different polarity between both ligands dictates a phase-separated ligand shell, as previously reported on Au NPs.^16,17^Figure 1g–i present the ITC-extracted thermodynamic parameters and the calculated linear combination. The pure C6SH(F) exhibits higher exothermicity and entropy loss relative to the hydrocarbon ligand, C6SH. The polar nature of the C–F bond induces stronger interchain interactions within the C6SH(F) ligand shell. In addition, it has been observed that fluor-rich ligands prevent the penetration of small molecules into the ligand shell, due to unfavorable interactions between the fluorocarbon and hydrocarbon molecules.^34−36^ Both effects suggest compact ligand packing, with increasing vdW interactions, leading to elevated exothermicity and entropy loss.

For all the investigated C6SH:C6SH(F) compositions, the measured Δ*H* and Δ*S* values mostly correlate with the linear combination (Figure 1g and h, respectively), while the measured Δ*G* values present positive deviations from the linear combination (Figure 1i), suggesting overall unfavorable mixing. The limited contact interfacial region between clusters of each ligand results in small deviations from the linear combination for Δ*H* and Δ*S*. These deviations demonstrate a slight reduction in the exothermicity and a small increase in the entropic loss, as expected for the unfavorable mixing at the interface. In contrast to the C6SH:C10SH mixture, all extracted thermodynamic parameters collectively point to an assembly pattern where, due to their different polarity, the ligands tend to fully segregate and form distinct domains of each ligand on the NC shell (Janus-like surface pattern, Scheme 1).

To gain further insight into the link between ligand–ligand interactions and the resulting shell organization, we analyzed the experimental findings using several mixing thermodynamic models. Because experiments show a nonzero mixing enthalpy, the simplest “ideal mixture” model can be excluded. Instead, the mixture can be treated using the “regular solution” model, where the mixing free energy, Δ*G*_mix_, can be expressed as
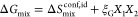
2

In eq 2, the first term on the right-hand side is the configurational entropy, Δ*S*_mix_^conf,id^, associated with the organization of the ligands on the NC surface and assumed to be ideal in this mean field model (SI, Section 6). The second term is the nonideal contribution, with ξ_G_ the free energy change involved in pairing two different ligands from pairs of similar ligands, which can have enthalpic and entropic components,
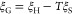
3

Thus, ξ_G_ embodies a nonideal mixing enthalpy Δ*H*_mix_ contribution and an additional nonconfigurational entropy, Δ*S*_mix_^nc^, associated with the intra- and interchain degrees of freedom, as was previously described for mixtures of long and short ligands^9,18^ (SI, Section 6).

For the C6SH:C10SH system, a good fit was achieved with ξ_H_ = 8 ± 3 kJ/mol (Figure 1d, blue line), resulting in a maximal value of Δ*H*_mix_ = 2 ± 1 kJ/mol, for *X*_C6SH_ = 0.5. Positive enthalpic interaction parameter ξ_H_ is consistent with a loss of interactions due to C6SH:C10SH pairing. The difference in measured enthalpy between the pure C6SH and C10SH ligands is 1 kJ/mol per carbon; thus the expected maximal loss of end-chain interactions for C10SH upon mixing with C6SH is 4 kJ/mol. Yet, we find a lower value for Δ*H*_mix_, indicating that either the C10SH ligands bend toward the NC surface,^21,37^ so as to compensate for the loss of end-chain interactions, and/or that inhomogeneous mixing is realized with partial ligand segregation (i.e., ligand clustering), leading to lower loss of interactions because of the smaller interfacial regions.

Considering Δ*S*_mix_^conf,id^ as the sole entropic term (i.e., setting ξ_S_ = 0) already provides a good fit to the ITC-extracted Δ*S* (Figure 1e, blue line), thus suggesting a well-mixed ligand shell. However, Δ*G*_mix_, derived as the sum of fitted Δ*H*_mix_ and Δ*S*_mix_, mostly results in positive deviations from the linear combination over most of the measured molar fraction range (Figure 1f, blue solid line). In the mean-field representation, Δ*G* for the ligand exchange reaction can then be determined by the common tangent construction (Figure 1f, blue dashed line). The region between the two contact points on the Δ*G* curve should correspond to meta- or unstable states that should evolve to phase separation between the two ligands. However, this result is inconsistent with the known similar polarity of both ligands and the observed gain in Δ*S*_mix_. Indeed, previous works on binary ligand shells of varying hydrocarbon lengths suggested some degree of ligand segregation, resulting in the formation of clusters on the NC surface (Scheme 1), but not full phase separation.^9^ This apparent inconsistency may be a consequence of the small size of the NC surface (127 surface sites), since finite size effects are unaccounted for in simple mean-field models.

To account for the shortcomings of mean-field representation, we used a Monte Carlo-based simulation with thermodynamic integration^38^ to numerically evaluate Δ*G*_mix_, Δ*H*_mix_, and Δ*S*_mix_ for a finite-sized system (Figure 1f, red, SI, Section 6). The model considers pairwise interactions χ_G_ between nearest neighbors on a square lattice. This interaction includes enthalpic and entropic components of the form
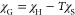
4

Fits to the experimental results yielded interaction parameters of χ_G_ = 3.4*RT*, χ_H_ = 15.4 kJ/mol, and χ_S_ = 23 J/mol K, from which Δ*H*_mix_ and total Δ*S*_mix_ were calculated (Figure 1d and e, respectively, red), while also accounting for the exact Δ*S*_mix_^conf^ (Figure 1e, black, SI, Section 6). Both χ_H_ and χ_S_ are positive, which points to enthalpically unfavorable mixing compensated by the entropic tendency to mix. However, the corrected conformational mixing entropy is lower than the ideal one (Δ*S*_mix_^conf^ < Δ*S*_mix_^conf,id^), suggesting partial ligand segregation and the formation of clusters. The same procedure applied to the C6SH:C6SH(F) binary set indicates a higher χ_G_ of 5.2*RT* (Figure 1i, red).

**Figure 2:**
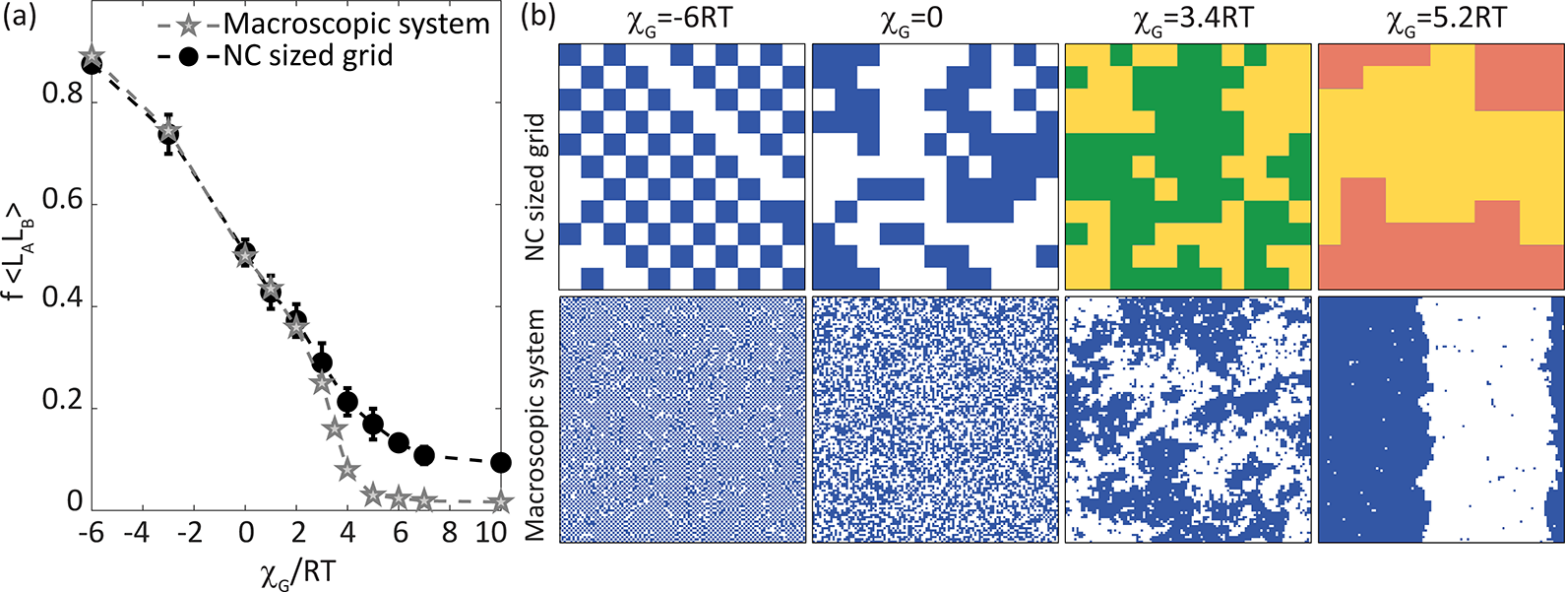
Comparison of ligand patterning in macroscopic and NC-sized simulations. (a) Average fraction of different ligand pairs (L_A_L_B_), as a function of the total interaction parameter, χ_G_, (normalized to the thermal energy *RT*). The molar fraction of surface ligands is *X* = 0.5. (b) Ligand shell organization for several χ_G_ values in the NC-sized grid (top) and the macroscopic system (bottom). Ligand shell patterning corresponding to interaction values for C6SH:C10SH and C6SH:C6SH(F) are presented in yellow and green, and yellow and orange, respectively.

Figure 2 compares simulation results for mixtures of two different ligands, L_A_ and L_B_, with molar fraction *X* = 0.5 and interaction parameter χ_G_ on the NC surface (11 × 11 = 121 sites) with a macroscopic system (111 × 111, similar results were achieved for a larger 1001 × 1001 grid) of the same composition. The black line, representing the NC system, does not show phase transition, but rather a continuous and gradual progression between different ligand surface patterning, as shown with insets. Zero interaction parameter (χ_G_ = 0) represents fully compensating enthalpy and entropy interaction parameters, so that the only driving force for mixing is the configurational entropy (Δ*G*_mix_ = −*T*Δ*S*_mix_ = −*T*Δ*S*_mix_^conf,id^), and the ligands distribute randomly with 50% of L_A_–L_B_ pairs on average. Note that even random mixing (compensating Δ*H*_mix_ and Δ*S*_mix_^nc^) should lead to some apparent clustering of ligands on the NC surface (Figure 2b). For the C6SH:C10SH mixture, where we find χ_G_ = 3.4*RT*, the enthalpic preference toward demixing overcomes the entropic preference toward mixing, and the resulting clusters are larger, with only 25% of L_A_–L_B_ pairs on average. This agrees with what has previously been observed for a binary shell with varying ligand lengths.^9^ Thus, compensation between the reduced enthalpy (loss of interchain interactions) and increased entropy dictates the extent of clustering.

For the C6SH:C6SH(F) mixtures, χ_G_ = 5.2*RT*, and mixing between the ligands is unfavorable. Here we find two well-separated regions of ligands with only 15% of L_A_–L_B_ pairs on average associated with the limited interface area. The opposite limit to the phase-separated ligand shell can potentially be achieved for negative values of χ_G_, leading to homogeneous (“checkerboard pattern”) organization driven by the favorable L_A_–L_B_ mixing, which we estimated at 90% L_A_–L_B_ pairs for χ_G_ = −6*RT*.

The larger influence of the interfacial fraction for small NC-like systems can be appreciated by comparing it to a macroscopic system approaching the thermodynamic limit. For the larger grid, a sharper change is observed between χ_G_ = 2*RT* and χ_G_ = 4*RT* (Figure 2a, gray stars), in agreement with the known critical point,^39,40^ where the L_A_–L_B_ pair fraction is expected to decrease abruptly with increasing χ_G_. Similarly, the average area of the simulated clusters swiftly changes around the critical point in the macroscopic system, while the simulated NC system presents only a moderate and gradual change (Figure 2b and Figure S13).

**Figure 3:**
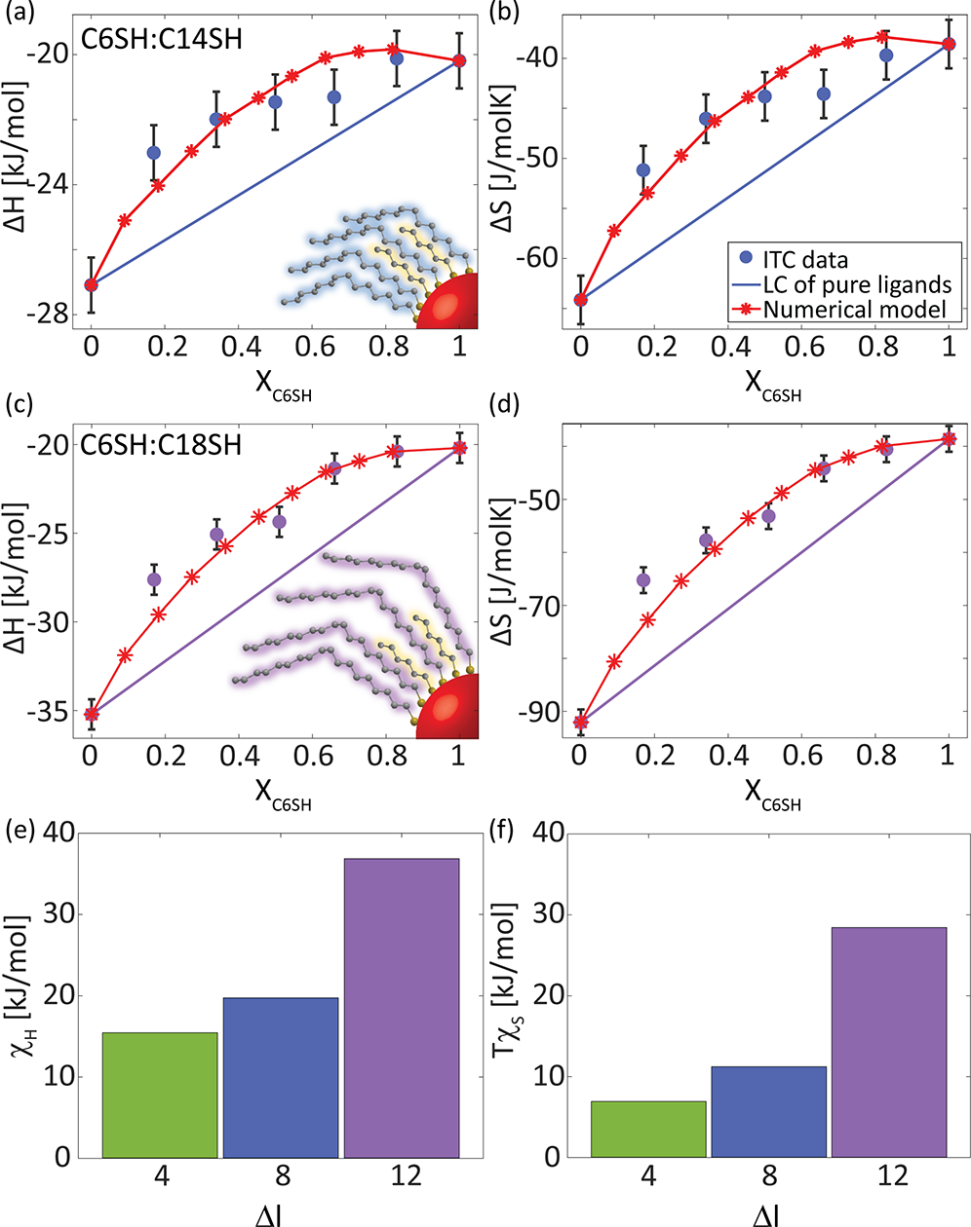
ITC-extracted (a, c) enthalpy and (b, d) entropy for the exchange reaction of oleate-coated CdSe NCs (*d* = 3.0 nm) with (a, b) C6SH:C14SH and (c, d) C6SH:C18SH binary compositions (0 < *X*_C6SH_ < 1, blue and purple dots, respectively). Blue and purple lines represent the linear combination (LC) of the pure ligands as a reference. Red asterisks and lines represent a fit to the numerical model. Inset: Illustration of the attached ligands. (e, f) Summary of the fitting-extracted (e) enthalpic and (f) entropic interaction parameters and their ligand length (Δ*l*) dependency.

The compensation between χ_H_ and χ_*S*_ was further studied for additional alkylthiol binary systems of a similar reference point, C6SH, with C14SH (Δ*l* = 8; Figure 3a,b) and with C18SH (Δ*l* = 12; Figure 3c,d). For both compositions, the extracted values for Δ*G* show minor deviations from the linear combination (Figure S10) and a similar χ_G_ to the one calculated for the C6SH:C10SH mixture (χ_G_ = 3.4*RT*). Hence, ligand clustering is also expected in the C6SH:C14SH and C6SH:C18SH mixtures. Similar trends of positive deviations from linear combination in Δ*H* and Δ*S* were observed. Yet, these deviations become larger as Δ*l* increases. The maximal deviation in Δ*H* increases from 1.8 through 3 to 5.5 kJ/mol as Δ*l* grows from 4 through 8 to 12, respectively. Correspondingly, χ_H_ is positive (endothermic mixing) and increases with increasing Δ*l*, due to a higher loss of end-chain interactions at the interfaces between both ligands (Figure 3e). Likewise, the maximal deviation in Δ*S* increases from 6 through 9.5 to 20 J/mol K as Δ*l* increases from 4 through 8 to 12, respectively. We find that the fitted positive χ_S_ compensates for the loss of interactions by increasing the entropic gain as Δ*l* grows due to the additional release of degrees of freedom for the longer ligands at the interfacial contacts (Figure 3f).

**Figure 4:**
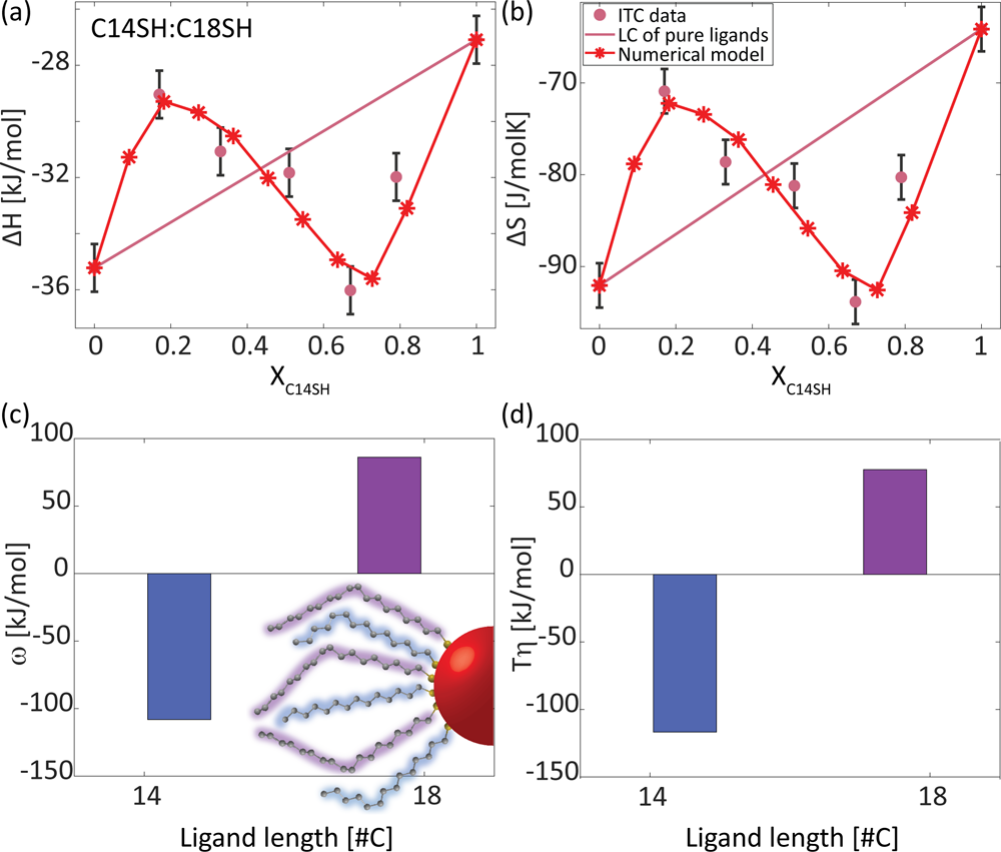
(a) ITC-extracted enthalpy and (b) entropy for the exchange reaction of oleate-coated CdSe NCs (*d* = 3.0 nm) with a mixture of C14SH and C18SH at different ratios (pink dots). The parameters of the pure ligands and their linear combination (LC) are presented for reference (pink line). The fitting of the ITC data with a numerical model is presented in red asterisks and lines (c) Fitting-extracted enthalpic and (d) entropic interaction parameter coefficient for both ligands. Inset: Illustration of the attached ligands.

Careful examination of the experimental results reveals slightly higher deviations from the linear combination and from the fit for low *X*_C6SH_, as was observed for the C6SH:C10SH system above. This asymmetric behavior is even more pronounced for mixing two long ligands: C14SH and C18SH (Figure 4). Unlike the C6SH:C10SH system with similar Δ*l* = 4, for which a monotonic behavior is observed upon changing *X*_C6SH_, for the C14SH:C18SH mixture, above a certain *X*_C14SH_ the contribution of mixing becomes exothermic (Figure 4a) with higher entropy loss (Figure 4b). For low *X*_C14SH_, the mixing enthalpy (or mixing entropy) is mainly affected by the loss of interactions (or increase in degrees of freedom) of the longer C18SH, which is the majority component. Therefore, mixing is endothermic with positive entropic contribution, as observed for the previously discussed systems. For high *X*_C14SH_, however, the short chain, which dominates the mixing characteristic, gains much more interactions when mixing with C18SH, thus forming a tightly packed ligand shell and consequently losing entropy through mixing. This is also potentially related to the previously observed tendency of long ligands to lie flat on the NC surface.^37^ The long C18SH better overlaps the C14SH chain, as both of them tend to fold.

To explain these experimental results, molar fraction-dependent interaction parameters are required. We define interaction parameters that depend linearly on the molar fraction of the components, similar to the ones suggested by the subregular solution model.^41,42^
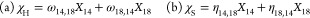
5where ω_*i*,*j*_ and η_*i*,*j*_ represent the change of enthalpy and nonconfigurational entropy, respectively, resulting from the insertion of ligand *j* to a region containing ligand *i*. The interaction parameters obtained from Equation 5 provide a better fit of the ITC-extracted Δ*H* and Δ*S* (Figure 4a and b, respectively), while Δ*G*_mix_ ≈ 0, as observed experimentally (Figure S10), allowing us to use the same χ_G_ = 3.4*RT*. Interestingly, the coefficients ω_14,18_ and η_14,18_ are negative (−108 kJ/mol and −117 J/mol K, respectively), while the coefficients ω_18,14_ and η_18,14_ are positive (86 kJ/mol and 78 J/mol K, respectively, Figure 4c and d). The sign change supports our conjecture regarding the gain in interactions and loss of nonconfigurational entropy for the shorter C14SH when in proximity to the longer C18SH, and vice versa for C18SH.

## Conclusions

Using ITC, we were able to follow the thermodynamics of ligand exchange reaction from oleate-coated NCs toward a binary composition of alkylthiols. Our findings revealed that binary compositions with similar polarity exhibited a compensation mechanism between enthalpy and entropy of mixing, leading to almost no change in free energy upon mixing. Using simple models, we resolved the entropic and enthalpic contributions due to ligand–ligand interactions, as well as the contribution of the configurational entropy on the NC surface. Larger differences between the mixed ligand chains resulted in higher mixing endothermicity, compensated by an increase in the mixing entropy. This trend is mostly due to the loss of interactions at the ligand–ligand contact interfacial zone between different ligand domains, which increases with increasing ligands’ length difference (Δ*l*). For systems with similar Δ*l* but longer ligand lengths, we find a nonmonotonic change in the mixing enthalpy and entropy, which we rationalize by a difference in the thermodynamic response of both ligands to changes in their surroundings. Based on the calculated total interaction parameter χ_G_ values, we could infer the mixed shell structure, which is most strongly affected by the chemical nature of the ligands (hydrocarbon vs fluorocarbon). Our findings demonstrate that in contrast to macroscopic surfaces, the small dimensions of the NCs and the subsequent increased interfacial region between nonsimilar ligands allow the formation of a myriad of clustering patterns, controlled by the interligand interactions. As a rule of thumb, we find that clustering is expected for a mixture of alkylthiol ligands with similar polarity but with different ligand length. By contrast, binding of mixed ligands with different polarities (hydrocarbon and fluorocarbon) but similar lengths resulted in their separation into segregated areas on the NC surface. Our findings aid in the fundamental understanding of the formation of surface ligand clustering versus phase-separated Janus-like surface arrangement, which should allow smarter surface design toward NC-based applications.

## Experimental Section

### Chemicals

1-Octadecene (90%), oleic acid (90%), CdO (≥99.99%), Se powder (100 mesh, 99.99%), trichloroethylene (anhydrous, ≥99%), 1-hexanethiol (97%), and 1-octadecanethiol (98%) were purchased from Sigma-Aldrich. 1-Decanethiol (96%) and 1-tetradecanethiol (94%) were purchased from Alfa Aesar. 1*H*,1*H*,2*H*,2*H*-Perfluoro-1-hexanethiol (97%) was purchased from Synquest Laboratories. Hydrochloric acid (37%) and HPLC grade hexane were purchased from Bio-Lab.

### Methods

Absorption measurements were performed using a JASCO V-770 UV–vis–NIR spectrophotometer. TGA measurements were performed using TGA-5500 (TA Instruments). ITC experiments were performed using a NanoITC calorimeter (TA Instruments) equipped with 1 mL of Hastelloy sample and reference cells with a 250 μL syringe. The ITC curves were fitted using NanoAnalyze Software v 3.10.0 (TA Instruments). Further data analyses including mixing model fitting and ligand shell structure calculations were done using MATLAB (The MathWorks Inc.).

### CdSe NCs Synthesis

CdSe NCs (*d* = 3 nm) in a zinc blende structure were synthesized by modifying a known procedure.^10,22^ Briefly, in a 100 mL three-neck flask 4 mL of 0.2 M Cd-oleate and 13 mL of ODE were degassed under vacuum at 100 °C for 1 h. Then the temperature was increased to 240 °C under Ar flow, and 4 mL of 0.1 M Se suspension in ODE was quickly injected. An additional 200 μL of 0.1 M Se-ODE suspension was injected every 5 min to avoid Ostwald ripening. The NCs reached the desired size (*d* = 3 nm) after 30 min. The NCs were precipitated from the synthesis crude solution by centrifugation with toluene and ethanol at 60K rpm for 10 min. Then, the NCs were redispersed in toluene and precipitated again with ethanol. The last step was repeated three times in order to get rid of excess ligands. The clean NC solution was kept in trichloroethylene (TCE) under an Ar atmosphere. The NC size was determined from a previously reported sizing curve based on the first exciton peak position (Figure S1).^43,44^

### ITC Measurements

Alkylthiol ligands and purified NCs dispersed in TCE were used for ITC measurements. This solvent was chosen due to its relatively high boiling point and a relatively low enthalpy of mixing with the ligands.^45^ In addition, all investigated ligands and NCs are well dispersed in TCE. The NCs’ concentration was determined from the solution absorption, based on a previous report of the extinction coefficient.^43^ The surface site concentration was calculated based on a simple spherical model of zinc blende CdSe with a lattice parameter of 6.050 Å (SI, Section 3). For each titration, 1 mL of NCs solution was injected to the ITC sample cell, and the ligand solution was loaded to the 250 μL ITC syringe. The surface sites and the ligand concentration were adjusted in order to produce high-quality titration curves. At each injection step 5 μL of ligands solution was injected to the cell, and the heat flow was measured for 600–800 s, during which the system returned to equilibrium. All ITC thermograms and exchange-model fitted titration curves, including detailed derivation of the single-site model, are presented in the SI, Section 4.

### Calculations of Thermodynamic Parameters for Mixing and Ligand Shell Structure

The ITC-extracted thermodynamic parameters were fitted to the sum of the linear combination and the calculated Δ*G*_mix_, Δ*H*_mix_, and Δ*S*_mix_ using the thermodynamic integration method. The binary shell structure was simulated based on a lattice model according to the interaction free energy parameter between the ligands, χ_G_. Generally, paring two different ligands is allowed with the Boltzmann probability , where  is derived directly from χ_G_ and the change in the ligand’s nearest neighbors due to the pairing process. In the first step of the fitting procedure, Δ*G*_mix_ is evaluated, while the temperature dependence of χ_G_ allowed calculating also Δ*H*_mix_ and Δ*S*_mix_. Further model details are in the SI, Section 6.

## Supporting Information Available

ITC raw data thermograms, titration curves and fitting to the exchange model, surface analysis via TGA, as well as a detailed explanation of the mixing models discussed in the text (PDF)
