# Supplementary material for: Spontaneous Patterning of Binary Ligand Mixtures on CdSe Nanocrystals: From Random to Janus Packing
Source: ACS Nano. 2023 Mar 9;17(6):5852–60. doi: 10.1021/acsnano.2c12676 (PMC10061916; doi:10.1021/acsnano.2c12676)
Supplement: Supplementary file 1 — nn2c12676_si_001.pdf [file nn2c12676_si_001.pdf]

`

Supporting Information

Spontaneous Patterning of Binary Ligand Mixtures on CdSe Nanocrystals: from Random to Janus Packing

Orian Elimelech,[a] Meirav Oded,[a] Daniel Harries,[a,b] and Uri Banin[a]**

a The Institute of Chemistry and The Center for Nanoscience and Nanotechnology, The Hebrew University of Jerusalem, Jerusalem 9190401, Israel.

b The Fritz Haber Center, The Hebrew University of Jerusalem, Jerusalem 9190401, Israel.

*E-mail: Uri.Banin@mail.huji.ac.il

Daniel.Harries@mail.huji.ac.il

`

Table of contents:

1. CdSe NCs synthesis 3

2. n-Alkylthiols reduction 3

3. Surface sites calculation 3

4. ITC measurements and analysis 6

4.1. Derivation of a single-site ligand exchange model 6

4.2. Experimental data and fitting 8

5. Additional surface characterization 16

6. Models of binary ligand shell 19

6.1 Ideal mixture model 19

6.2 Regular mixture model 19

6.3 Resolving thermodynamic parameters using thermodynamic integration 20

1. CdSe NCs synthesis

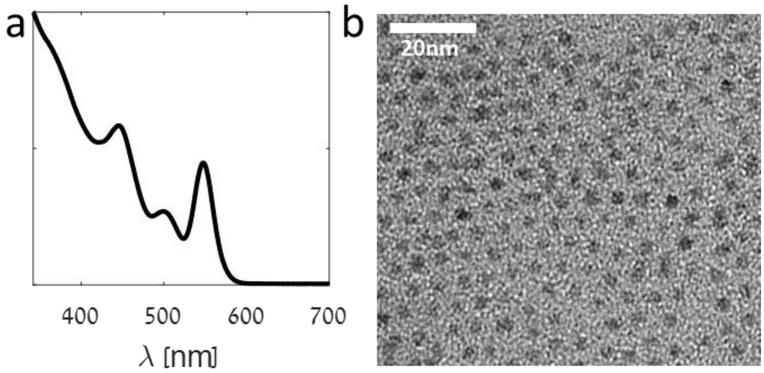

Figure S1. *(a) Absorption spectra and (b) TEM image of as-synthesized oleate coated CdSe NCs.*

2. n-Alkylthiols reduction

To avoid inaccuracies in the ligands concentration, derived by S-S chain coupling bonds, the purchased ligands were reduced prior to use, as was discussed in detailed elsewhere.[1]. Briefly, two equivalents of $NaBH_4$ powder were added to a solution of alkylthiol in ethanol and TDW (1:4). After 12 hours of stirring at room temperature, the solution was extracted with chloroform (3x75 ml portions). Following that, the unified organic phase was dried over $MgSO_4$, filtered and then evaporated under vacuum in order to separate between the chloroform and the reduced alkylthiol. The reduced ligands were kept under an inert atmosphere with no exposure to UV light for future use. The yield of the reduction procedure is 70%, to give a final product with no more than 5% of disulfide.

3. Surface sites calculation

As described in our previous studies,[1,2] the number of Cd surface sites ($N_{surface}$) was calculated based on a simple spherical model for the NCs with a lattice parameter of a=6.050Å (zinc blende). We assumed a uniform zinc blende CdSe layer on the surface, hence the number of Cd surface sites is

$$\text{(S1)} \qquad N_{surface} = N_{total} - N_{internal-sphere}$$

where N_{total} is the total number of Cd atoms in the NC and $N_{internal\text{-}sphere}$ is the number of core Cd. N_{total} was calculated considering the volume of a spherical NC with a radius R_{NC}, the density of CdSe (ρ_{CdSe}) and its molar mass (Mw_{cdSe}):

$$\text{(S2)} \qquad N_{total} = \frac{\frac{4}{3}\pi R_{NC}^3 \cdot \rho_{CdSe}}{Mw_{CdSe}} \cdot N_A$$

`

$N_{internal\text{-}sphere}$ was calculated in a similar way to N_{total} excluding the outer layer of the surface Cd:

(S3) $$N_{internal-sphere} = \frac{\frac{4}{3}\pi\left(R_{NC}-\frac{a}{2}\right)^3 \cdot \rho_{CdSe}}{Mw_{CdSe}} \cdot N_A$$

For the investigated d=3.0 nm NC, 127 Cd surface sites are expected.

The results were compared with a pyramidal model for zinc blend CdSe NCs with four exposed (111) facets. The height of the pyramid, *h,* was taken as the calculated diameter of the NC, hence the edge length, *c*, is:

(S4) $$c = \sqrt{\frac{3}{2}}h$$

The Cd atoms are spaced on the edge according to the nearest-neighbor distance, *d*, of the unit cell:

(S5) $$d = \frac{\sqrt{2}}{2}a$$

Hence, the length of an edge, *c*, containing *N* atoms is:

(S6) $$c = \frac{1}{\sqrt{2}}a(N-1)$$

Using eq. (S4) in eq. (S6), we can determine the number of atoms on the edge:

(S7) $$N = \frac{h\sqrt{3}}{a} + 1$$

Since the zinc blend NCs are actually a truncated pyramid, the atoms of the outer edges were removed, and the new faces lost one atoms per line, per side. Therefore, the number of Cd surface atoms on a single face, N_{face}, with a base containing *(N-2)* atoms is calculated by:

(S8) $$N_{face} = \sum_{q=1}^{N-2} q = \frac{(n-2)(n-2+1)}{2}$$

By using eq. (S7) in eq. (S8) and multiplying it by 4 (for the four faces), we find the total number of Cd surface atoms in all four faces as a function of the pyramid height:

(S9) $$N_{surface} = 2\left(\frac{h\sqrt{3}}{a}+1\right)^2 - 6\left(\frac{h\sqrt{3}}{a}+1\right) + 4$$

For the investigated d=3.0nm NC, 130 Cd surface sites are expected.

In addition, an atomistic model was also considered to verify the suggested models for surface sites. A semi-spherical NC was simulated from the bulk zinc blend CdSe crystal structure by removing atoms located beyond a distance that is greater than the desired radius (Figure S2). The remaining atoms resulted in a non-stoichiometric ratio between Cd and Se atoms. The atoms in the outer layer were considered as surface sites. To account for the range of error in the size estimation, results for NC diameters of 2.8, 3.0 and 3.2 nm are presented in Table S1. For an average d=3.0±0.2 nm NC (similar to the experimentally extracted distribution of the investigated NCs,), 128±47 Cd surface sites are expected with an average Cd:Se ratio of

1.1±0.1. The non-stoichiometric ratio we find is consistent with previous reports on CdSe NCs.[3]

All presented models give similar average numbers of Cd surface sites.

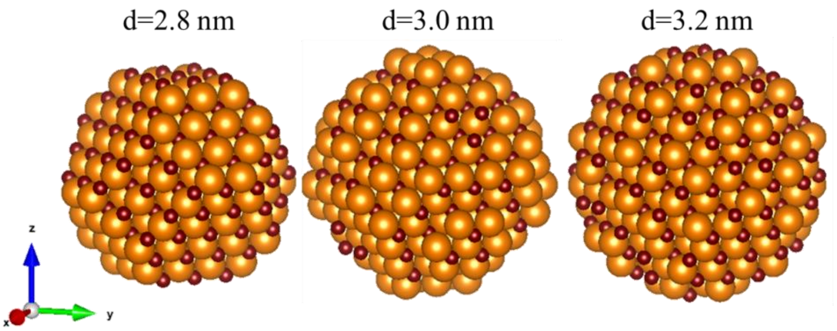

Figure S2. *Simulated atomistic model for zinc blend CdSe NC of diameters 2.8, 3.0 and 3.2 nm. Cd and Se atoms are colored brown and orange respectively.*

NC diameter	Cd atoms	Cd:Se ratio	Surface Cd	Surface Se
**2.8**	201	1.14	114	96
**3.0**	225	0.94	90	100
**3.2**	321	1.16	180	124

Table S1. *Summary of the simulated atomistic model data for zinc blend CdSe NC of diameters 2.8, 3.0 and 3.2 nm, as presented in Figure S2.*

`

4. ITC measurements and analysis

4.1. Derivation of a single-site ligand exchange model

As described in our previous studies,[1,2] the ligand exchange model is based on the well-known "single set of independent binding sites" model.[4] We modified the known "binding" model, which considers only the attachment of the new ligand, in order to take into account also the detachment of the native ligand. This "exchange" model is necessary for ligand exchange reactions since both processes, detachment and attachment of the ligands, release heat which is measured by the ITC instrument.

The ligand exchange reaction between the native ligand L' and the exchanged ligand L for a single surface site M is

(S10) $ML' + L \rightleftharpoons ML + L'$

The equilibrium constant is defined as

(S11) $K = \frac{[ML][L']}{[ML'][L]}$

Assuming that each native ligand L' is exchanged with a single new ligand L and no free L' is present initially[5] (supported by TGA data, see in the next section), we get:

(S12) $[ML] = [L']$

While for the exchanged ligands:

(S13) $[L] = [L]_0 - [ML]$

And for all surface sites:

(S14) $[ML'] = n[M]_0 - [ML]$

In the previous equations, $[M]_0$ is the total number of surface sites on the NC (based on a spherical model, as explained before), n is the ratio between the actually exchanged ligands and the available surface sites (i.e., the reaction stoichiometry coefficient), and $[L]_0$ is the total added exchanged ligand.

Given the expressions above, the equilibrium constant can be written as:

(S15) $K = \frac{[ML]^2}{(n[M]_0 - [ML])([L]_0 - [ML])}$

We define θ as the NC surface coverage, and hence,

(S16) $[ML] = n[M]_0\theta.$

Given eq. (S12), eq. (S15) can be rewritten as

(S17) $0 = \theta^2 - \theta\left(\frac{K}{K-1}\right)\left(1 + \frac{[L]_0}{n[M]_0}\right) + \left(\frac{K}{K-1}\right)\left(\frac{[L]_0}{n[M]_0}\right)$

During an ITC experiment, we measure the total amount of heat released per injection of ligand, which is correlated with the enthalpy change of the reaction

`

(S18) $Q_{total} = \theta n[M]_0 V_{cell} \Delta H$

By using the solution for the quadratic equation(S17), eq. (S18) can be written as

(S19) $$Q_{total} = \frac{n[M]_0 V_{cell} \Delta H}{2}\left[\left(\frac{K}{K-1}\right)\left(1+\frac{[L]_0}{n[M]_0}\right) - \sqrt{\left(\frac{K}{K-1}\right)^2\left(1+\frac{[L]_0}{n[M]_0}\right)^2 - 4\left(\frac{K}{K-1}\right)\left(\frac{[L]_0}{n[M]_0}\right)}\right]$$

and the heat released per injection of ligand is

(S20) $$\frac{dQ_{tot}}{d[L]_0} = \frac{V_{cell} \Delta H}{2}\left(\frac{K}{K-1}\right)\left[1 - \frac{\frac{[L]_0}{n[M]_0}+\frac{2-K}{K}}{\sqrt{1+\left(\frac{[L]_0}{n[M]_0}\right)^2+\left(\frac{[L]_0}{n[M]_0}\right)\left(\frac{4-2K}{K}\right)}}\right]$$

where

(S21) $$d[L]_0 = \frac{V_{injection}[L]_{syringe}}{V_{cell}}$$

By implanting eq. (S21) into eq. (S20), we get the final equation for fitting

(S22) $$dQ_{tot} = \frac{V_{injection}[L]_{syringe} \Delta H}{2}\left(\frac{K}{K-1}\right)\left[1 - \frac{\frac{[L]_0}{n[M]_0}+\frac{2-K}{K}}{\sqrt{1+\left(\frac{[L]_0}{n[M]_0}\right)^2+\left(\frac{[L]_0}{n[M]_0}\right)\left(\frac{4-2K}{K}\right)}}\right]$$

The other thermodynamics parameters ΔG and ΔS are calculated by using the known thermodynamics relations

(S23) $\Delta G = -RT \ln K$

(S24) $\Delta S = \frac{\Delta H - \Delta G}{T}$

`

4.2. <u>Experimental data and fitting</u>

All fittings were done in NanoAnalyze Software v 3.10.0 (TA instrument).

4.3.1. <u>Error analysis:</u>

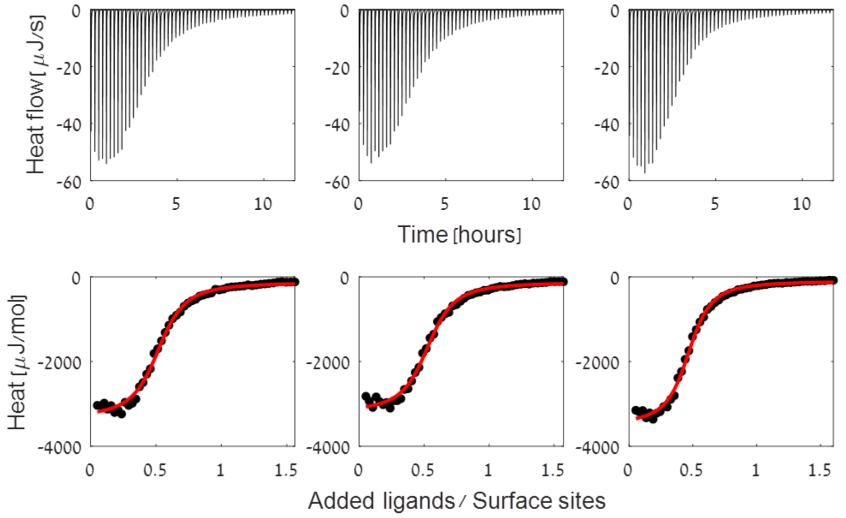

Figure S3. *Real-time thermograms and the corresponding titration curves with their model fitting for the ligand exchange reaction of oleate-coated CdSe NCs with an equimolar mixture (1:1) of C6SH and C14SH at 303K.*

	**Ligand [mM]**	**Surface sites [mM]**	**ΔH [kJ/mol]**	**ΔS [J/molK]**	**ΔG [kJ/mol]**	**n**
**Exp. 1**	30	5.7±0.2	-23.2±0.7	-48±5	-8.5±0.4	0.49±0.03
**Exp. 2**	29	5.6±0.2	-22.4±0.7	-47±5	-8.0±0.4	0.55±0.03
**Exp. 3**	30	5.8±0.2	-21.5±0.7	-44±4	-8.2±0.4	0.56±0.03

Table S2. *The thermodynamic parameters extracted from the model fit of the titration curves for the ligand exchange reaction of oleate-coated CdSe NCs with an equimolar mixture (1:1) of C6SH and C14SH at 303K, presented in Figure S3. Errors were calculated based on the quality of the fitting.*

Errors of the extracted thermodynamics parameters were determined by the quality of the fitting. In addition, we considered the reproducibility of the measurement by performing the same experiments three times and calculating the standard deviation of each parameter.

The error in the surface site's concentration was calculated by a triple measurement of the absorption.

4.3.2. ITC data and analysis for ligand exchange with pure alkylthiols

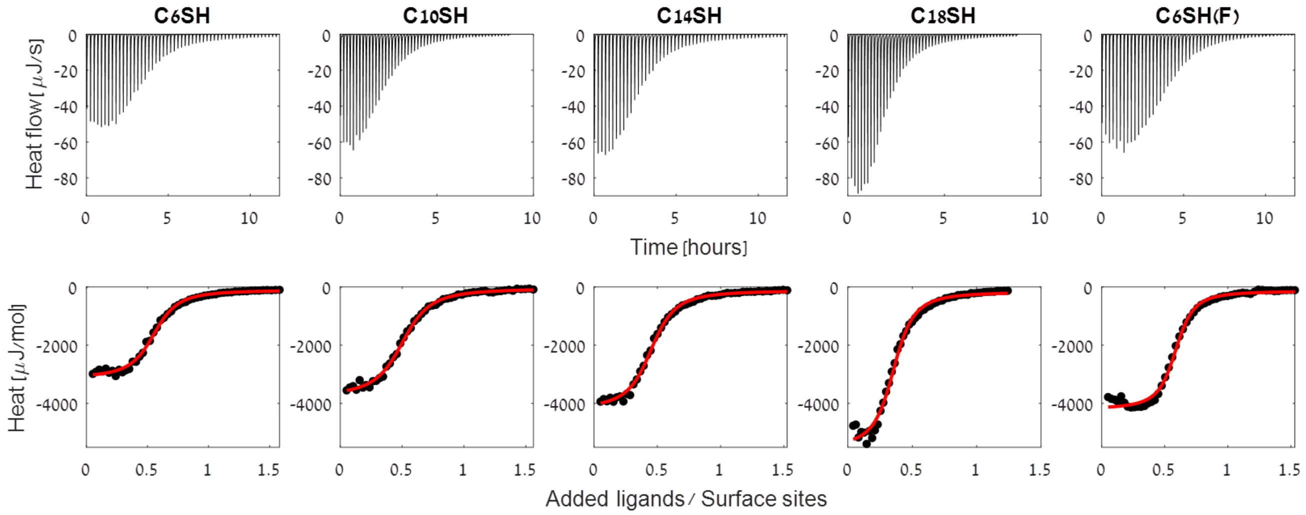

Figure S4. *Real-time thermograms and the corresponding titration curves with their model fittings (red) for the ligand exchange reaction of oleate-coated CdSe NCs with pure alkylthiols at 303K.*

**Ligand**	**Ligand [mM]**	**Surface sites [mM]**	**ΔH [kJ/mol]**	**ΔS [J/molK]**	**ΔG [kJ/mol]**	**n**
**1-Hexanethiol (C6SH)**	31	5.9	-20.2	-39	-8.5	0.58
**1-Decanethiol (C10SH)**	30	5.8	-24.3	-56	-7.5	0.54
**1-Tetradecanethiol (C14SH)**	31	6.1	-27.1	-64	-7.6	0.48
**1- Octadecanethiol (C18SH)**	23	5.7	-35.2	-92	-7.3	0.46
**1H,1H,2H,2H-Perfluoro-1-hexanethiol (C6SH(F))**	30	5.9	-27.5	-56	-10.4	0.59

Table S3. *A table summarizing the thermodynamic parameters extracted from the single-site model fit of the titration curves for the ligand exchange reaction of oleate-coated CdSe NCs with pure alkylthiol at 303K, presented in Figure S4.*

`

4.3.3. ITC data and analysis for ligand exchange with a mixture of alkylthiols

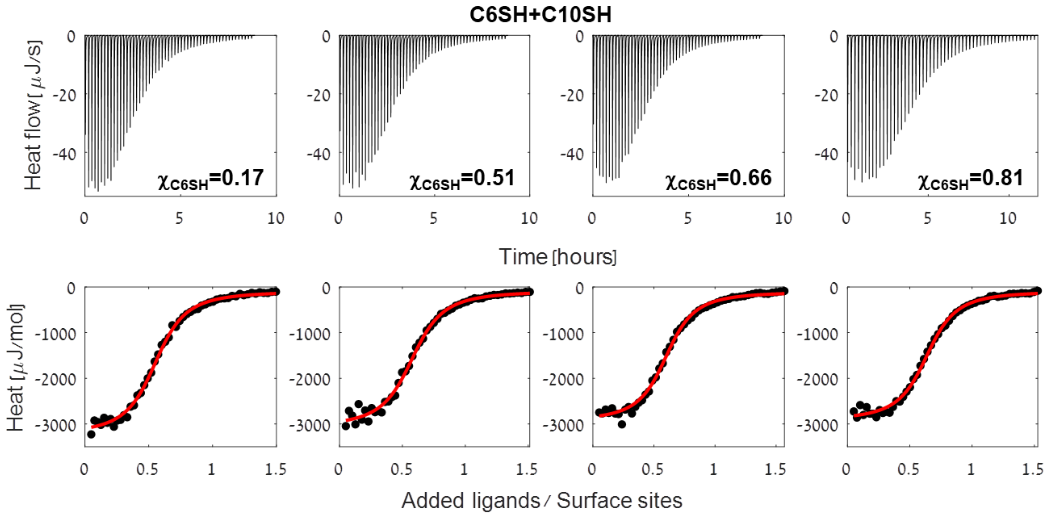

Figure S5. *Real-time thermograms and the corresponding titration curves with their model fittings (red) for the ligand exchange reaction of oleate-coated CdSe NCs with different mixtures of C6SH and C10SH at 303K.*

χ(C6SH)	Ligand [mM]	Surface sites [mM]	ΔH [kJ/mol]	ΔS [J/molK]	ΔG [kJ/mol]	n
**0.17**	29	5.9	-21.9	-47	-7.7	0.59
**0.51**	30	6.1	-20.4	-41	-7.9	0.62
**0.66**	28	5.5	-19.9	-38	-8.2	0.63
**0.81**	29	5.7	-20.1	-39	-8.3	0.67

Table S4. *A table summarizing the thermodynamic parameters extracted from the single-site model fit of the titration curves for the ligand exchange reaction of oleate-coated CdSe NCs with different mixtures of C6SH and C10SH at 303K, presented in Figure S5.*

`

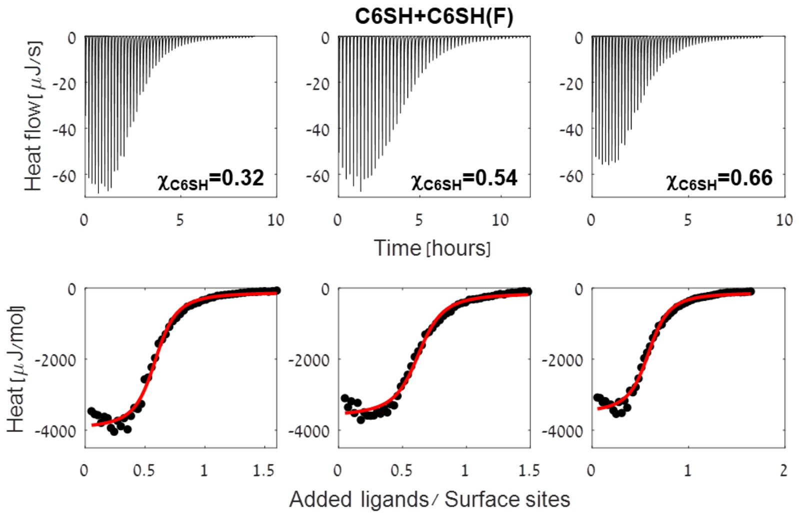

Figure S6. *Real-time thermograms and the corresponding titration curves with their model fittings (red) for the ligand exchange reaction of oleate-coated CdSe NCs with different mixtures of C6SH and C6SH(F) at 303K.*

**χ(C6SH)**	**Ligand [mM]**	**Surface sites [mM]**	**ΔH [kJ/mol]**	**ΔS [J/molK]**	**ΔG [kJ/mol]**	**n**
**0.32**	31	5.9	-25.3	-52	-9.3	0.60
**0.54**	30	6.1	-23.5	-48	-8.8	0.64
**0.66**	30	5.4	-22.1	-45	-8.5	0.61

Table S5. *A table summarizing the thermodynamic parameters extracted from the single-site model fit of the titration curves for the ligand exchange reaction of oleate coated CdSe NCs with different mixtures of C6SH and C6SH(F) at 303K, presented in Figure S6.*

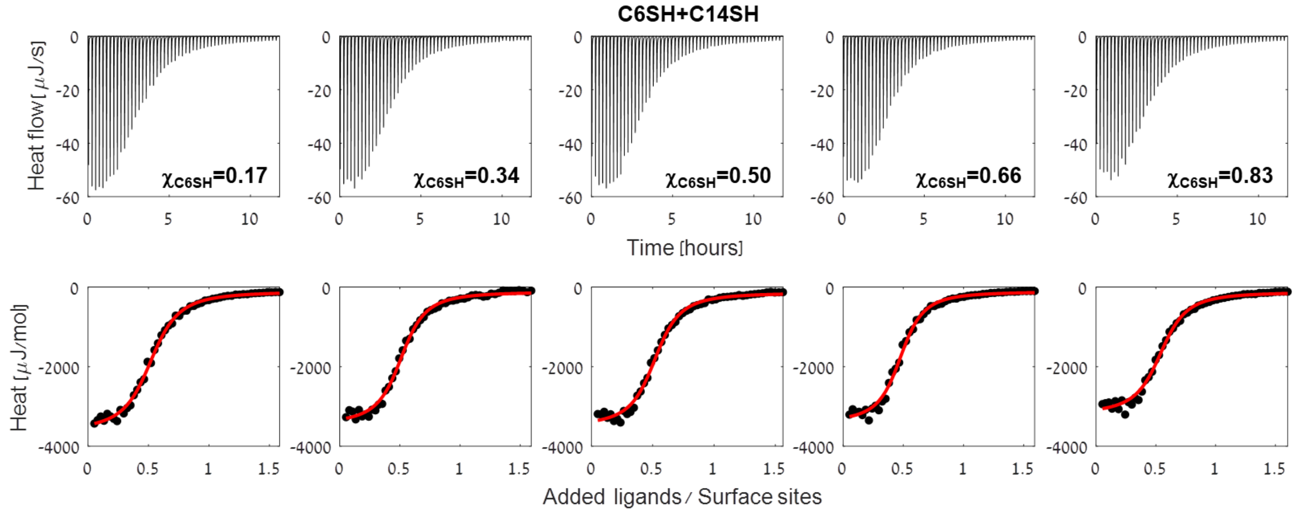

Figure S7. *Real-time thermograms and the corresponding titration curves with their model fittings (red) for the ligand exchange reaction of oleate coated CdSe NCs with different mixtures of C6SH and C14SH at 303K.*

**χ(C6SH)**	**Ligand [mM]**	**Surface sites [mM]**	**ΔH [kJ/mol]**	**ΔS [J/molK]**	**ΔG [kJ/mol]**	**n**
**0.17**	31	5.8	-23.0	-51	-7.5	0.56
**0.34**	30	5.8	-22.0	-46	-8.0	0.54
**0.50**	30	5.8	-21.5	-44	-8.2	0.56
**0.66**	30	5.8	-21.3	-43	-8.1	0.51
**0.83**	30	5.7	-20.1	-39	-8.1	0.57

Table S6. *A table summarizing the thermodynamic parameters extracted from the single-site model fit of the titration curves for the ligand exchange reaction of oleate-coated CdSe NCs with different mixtures of C6SH and C14SH at 303K, presented in Figure S7.*

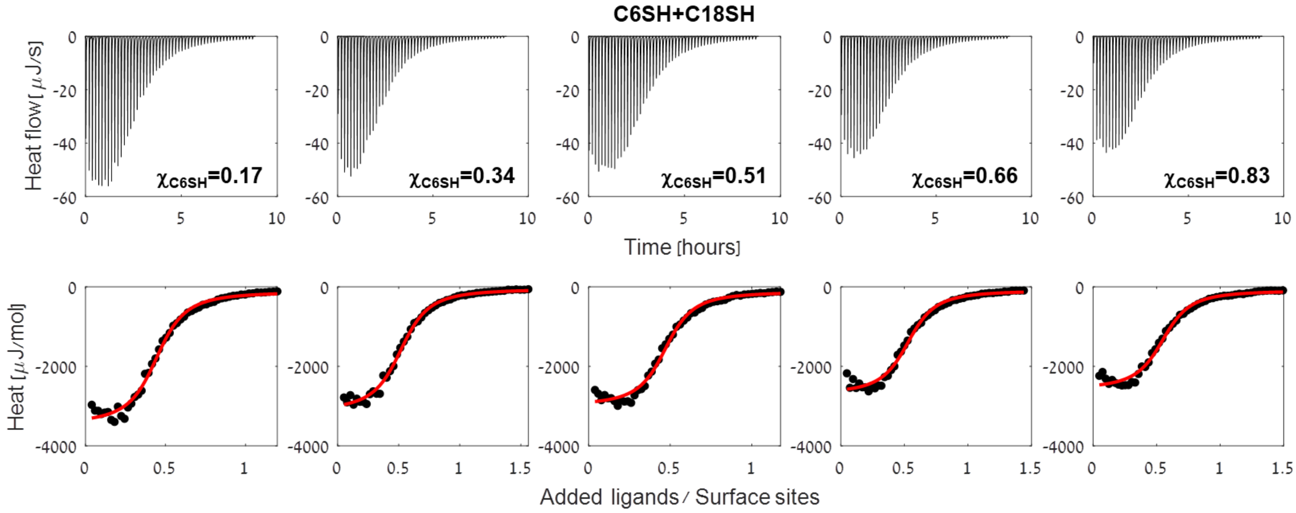

Figure S8. *Real-time thermograms and the corresponding titration curves with their model fittings (red) for the ligand exchange reaction of oleate-coated CdSe NCs with different mixtures of C6SH and C18SH at 303K.*

**χ(C6SH)**	**Ligand [mM]**	**Surface sites [mM]**	**ΔH [kJ/mol]**	**ΔS [J/molK]**	**ΔG [kJ/mol]**	**n**
**0.17**	25	6.3	-27.6	-635	-7.8	0.47
**0.34**	31	6.0	-25.1	-58	-7.6	0.56
**0.51**	25	6.3	-24.4	-53	-8.2	0.49
**0.66**	29	6.0	-21.3	-44	-8.0	0.56
**0.83**	30	6.1	-20.4	-40	-8.1	0.58

Table S7. *A table summarizing the thermodynamic parameters extracted from the single-site model fit of the titration curves for the ligand exchange reaction of oleate-coated CdSe NCs with different mixtures of C6SH and C18SH at 303K, presented in Figure S8.*

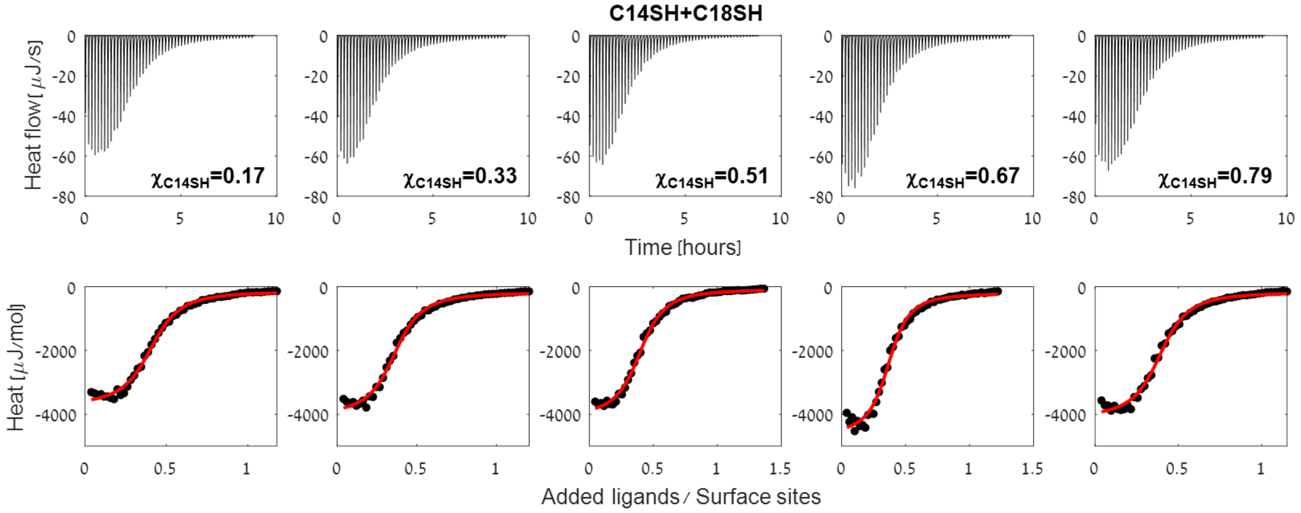

Figure S9. *Real-time thermograms and the corresponding titration curves with their model fittings (red) for the ligand exchange reaction of oleate-coated CdSe NCs with different mixtures of C14SH and C18SH at 303K.*

**χ(C14SH)**	**Ligand [mM]**	**Surface sites [mM]**	**ΔH [kJ/mol]**	**ΔS [J/molK]**	**ΔG [kJ/mol]**	**N**
**0.17**	25	6.5	-29.0	-71	-7.5	0.44
**0.33**	23	5.8	-31.1	-79	-7.2	0.39
**0.51**	22	5.0	-31.8	-81	-7.2	0.44
**0.67**	23	5.7	-36.0	-94	-7.6	0.39
**0.79**	23	6.1	-32.0	-80	-7.6	0.42

Table S8. *A table summarizing the thermodynamic parameters extracted from the single-site model fit of the titration curves for the ligand exchange reaction of oleate-coated CdSe NCs with different mixtures of C14SH and C18SH at 303K, presented in Figure S9.*

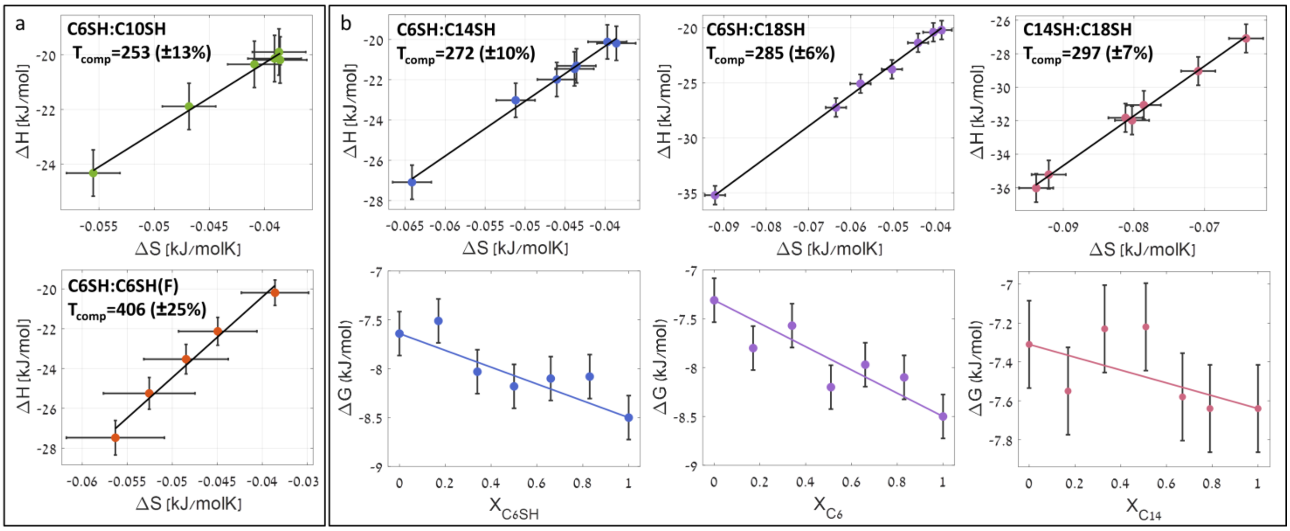

Figure S10. *(a) Enthalpy-entropy compensation (EEC) plot for C6SH:C10SH (top) and C6SH:C6SH(F) (bottom) binary compositions, according to the ITC results presented in Table S4 and Table S5, respectively). The compensation temperature (T_{comp}) in the C6SH:C6SH(F) system deviates from the experimental temperature (303K), correlating with the changes in the measured ΔG values which indicate on poor compensation (Figure 1 in the main text). (b) EEC plot (top) and ΔG experimental values (dots) vs. the calculated LC (line), for the investigated C6SH:C14SH (blue), C6SH:C18SH (purple), C14SH:C18SH (pink) binary systems. The observed EEC is correlated with the minor changes observed in ΔG. The ITC-extracted values of ΔG correspond with the LC values, considering the errors.*

`

5. Additional surface characterization

Thermogravimetric analysis (TGA) was used to quantify the changes in the organic coverage of the NCs upon ligand exchange, where the differences in the overall mass loss and in the shape of the TGA thermograms indicate the changes in the surface ligand layer composition. Representative TGA results for the NCs with their native oleate ligands and upon ligand exchange with pure C6SH, pure C10SH as well as with $C6SH_{0.5}$:$C10SH_{0.5}$ and $C6SH_{0.66}$:$C10SH_{0.34}$ binary compositions are presented in Figure S11. The initial oleate coverage of the NCs was determined via the mass loss up to 500°C, which is attributed to any organic species present in the sample. According to the thermogram, 44% of the total mass was organic, consistent with a full surface coverage and a 1:1 binding ratio (all Cd surface sites are bound to a single oleate ligand).

The post-ITC samples were analyzed similar to our previous report.[2] Prior to the analysis, the post-ITC samples were purified from excess free ligands by multiple cycles of precipitation and re-dispersion process using toluene (solvent) and ethanol (anti-solvent). As mentioned elsewhere,[2] the ITC conditions allow only partial ligand exchange, due to the small amounts of alkylthiol ligands added to the NCs in each titration point, resulting in an overall excess of alkylthiols to oleate of 1.5-2, which is insufficient to induce complete ligand exchange. This stands in contrast to the conditions for full ligand exchange which require an immediate disturbance to the system by the quick addition of a large excess of the exchanging ligand. Hence, the final surface coverage is slightly more complex, as more than one ligand type is involved. As can be observed from Figure S11, for the oleate coated NCs (red), the main mass loss is above 310°C, while the mass loss of the alkylthiolate in the post-ITC samples is mostly lower than that. Thus, similar to our previous report, the thermograms of the post-ITC samples were divided into 2 regions: 110-315°C, which is attributed to bound alkylthiolate (Figure S11, blue region), and 315-500°C, which is attributed to bound oleate (Figure S11, red region). The C6SH:C10SH ligand ratio in the binary shell samples was taken as the ratio in added ligand solution. Additional analysis for the binary shell composition is discussed in details in the next paragraph. The ligand composition results for all post-ITC samples are summarized in Table S9. According to the calculated fraction of each ligand, 82%±2 of the total ligand coverage is alkylthiolate and 18%±2 is oleate. We note that all TGA analysis can have up to 10% of inaccuracy due to the different purification efficiency of each sample. Considering this error and the ligand composition calculation, we conclude that exchange with ligand mixtures sparsely affect the final shell coverage and the exchange ratio. Moreover, since our

thermodynamic analysis focuses on comparison between different ITC measured samples (including the comparison of the ligand exchange with binary mixtures with the linear combination of the pure ligands parameters), we can discard the thermodynamic effect of oleate remains on the ligand shell, as it present in all samples with similar fractions.

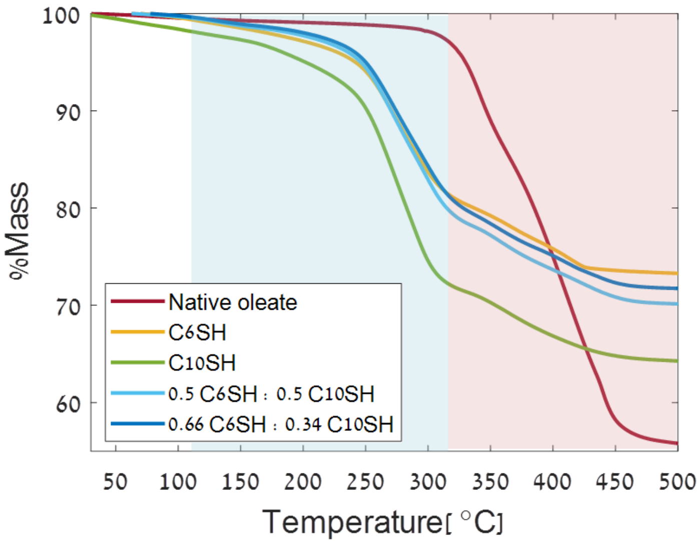

Figure S11. *TGA thermograms of purified CdSe NCs before (red) and after ITC-preformed ligand exchange with pure C6SH (yellow), pure C10SH (green) and* $C6SH_{0.5}$*:*$C10SH_{0.5}$ *(light blue) and* $C6SH_{0.66}$*:*$C10SH_{0.34}$ *(dark blue) mixtures. The differences in the temperature of inflection points and the loss of organic mass, indicate the changes in the surface ligands.*

Surface ligand	%Thiolate (110-315 ºC)	%Oleate (315-500 ºC)	Total coverage
**C6SH**	80%	16%	96%
**C10SH**	90%	17%	107%
**0.5 C6SH : 0.5 C10SH**	74%	19%	94%
**0.66 C6SH : 0.34 C10SH**	72%	19%	91%

Table S9. *Post-ITC ligand composition, as calculated from the TGA results presented in Figure S11.*

As described in the main text, the ITC data analysis in this work assumes that the ligand ratio on the NCs was as in the titrant added to the solution. To estimate the ligand ratio on the NC surface, we used the Langmuir model for competitive adsorption.[6] According to this model, the ratio of the surface absorbed molecules can be derived using ΔG values extracted from the experimental ITC curves. Specifically, the surface coverage of each component is given by:

. According to this model, we can estimate the ratio of the surface absorbed molecules, based on the ΔG values extracted from the experimental ITC curves. In this model. The surface coverage of each component is given by:

$$\text{(S25)} \quad \theta_A = \frac{K_{eq,A}X_A^{sol}}{1+K_{eq,A}X_A^{sol}+K_{eq,B}X_B^{sol}} \; ; \; \theta_B = \frac{K_{eq,B}X_B^{sol}}{1+K_{eq,A}X_A^{sol}+K_{eq,B}X_B^{sol}}$$

where $K_{eq,i}$ is the equilibrium constant for the exchange with pure ligand i (A or B, extracted from the ITC measurements with one ligand) and X_i^{sol} is the molar fraction of ligand i, added to the solution. Based on eq. (S25) we calculated the molar fraction of each ligand on the NC surface. Results for C6SH:C10SH binary compositions are shown in Figure S12, as a representative system. The observed deviation between the molar fraction added to the solution (green) and that estimated on the NC surface (grey) is minor. Hence, we have validated using the titrant's molar fraction in the numerical model used for extracting the interaction parameters between the ligands (mentioned in the main text and in the next section).

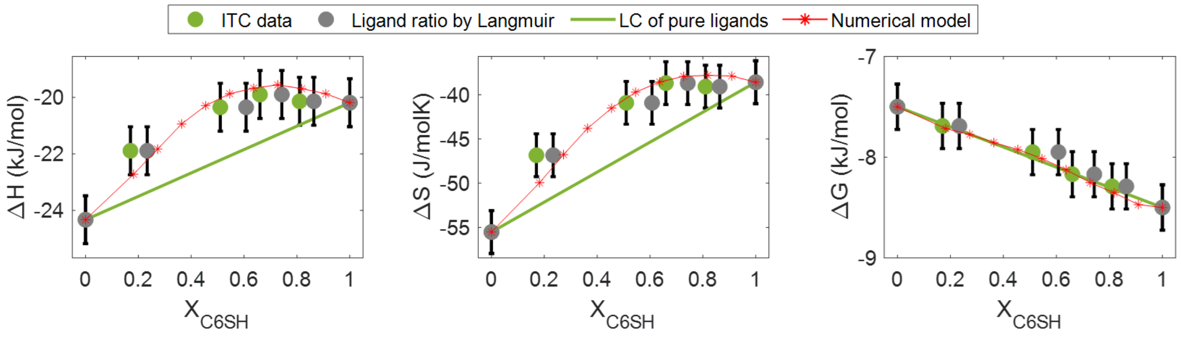

Figure S12. *ITC extracted thermodynamic parameters: enthalpy, entropy, and Gibbs free energy, for the exchange with C6SH (XC6SH=1), C10SH (XC6SH=0) and their binary composition (0<XC6SH<1). Data are presented as a function of C6SH molar fraction in solution (green) and on NC surface based on a competitive Langmuir model calculation (grey). Solid green line represents the calculated linear combination (LC) of the pure ligands. Red asterisk and line represent the numerical model.*

`

6. Models of binary ligand shell

6.1 Ideal mixture model

We define an ideal mixture as one where there is no excess enthalpy upon mixing ($\Delta H_{mix}=0$), and the mixing process is spontaneous due only to the increase in the configurational entropy. The configurational entropy is based on the enumeration of all the available states for spatially organizing the mixture. For an ideal binary mixture on a lattice with total L_A+L_B sites (L_A and L_B are the numbers of each ligand), the ideal configurational entropy of mixing is:

(S26) $$\Delta S_{mix}^{conf,id} = R\ln\frac{(L_A+L_B)!}{L_A!L_B!} \approx -R(L_A+L_B)\left(X_{L_A}\ln X_{L_A} + X_{L_B}\ln X_{L_B}\right)$$

where X_{LA} and X_{LB} are the molar fractions of the two components.

6.2 Regular mixture model

The regular mixture model is the simplest mean field model that allows to describe non-ideal mixing between two or more components. The model assumes an excess enthalpy and entropy of mixing (ΔH_{mix} and ΔS_{mix}, respectively) that are determined by the averaged environment of the components in the mixture. The excess enthalpy is modeled using an interaction parameter ξ_H that indicates the invested or released energy involved with pairing two nearest neighbors of different ligands from pairs of similar ligands:

(S27) $$\Delta H_{mix} = \xi_H X_{L_A} X_{L_B}$$

The excess entropy is determined by a configurational entropy that is approximated as the ideal contribution, eq.(S26), and a non-configurational entropy that includes an entropic interaction parameter ξ_s that indicates the corresponding change in intra and inter-ligand degrees of freedom upon ligands pairing:

(S28) $$\Delta S_{mix}^{nc} = \xi_S X_{L_A} X_{L_B}$$

The total free energy of mixing ΔG_{mix} is the sum of excess enthalpy and entropy (eqs. (S26)-(S28)):

(S29) $$\Delta G_{mix} = \Delta H_{mix} - T\left(\Delta S_{mix}^{conf,id} + \Delta S_{mix}^{nc}\right)$$

`

6.3 Resolving thermodynamic parameters using thermodynamic integration

Applying the regular solution mean field model resulted in an inconsistency in the expected ligand shell structures: while the similar chemical nature of the binary ligands (C6SH and C10SH) suggests some degree of mixing, the model-calculated Gibbs free energy values suggested phase separation. In order to resolve this discrepancy, we fit our data to numerically exact solutions of the model as applied to a small system.

The free energy of a binary system is calculated considering the interactions between all nearest-neighboring ligand pairs L_iL_j, according to the Hamiltonian:

(S30) $\mathcal{H} = \sum_{i,j}\left(\chi_{G,i,j}L_iL_j\right)$

where the sum is performed over all nearest neighbors and the inter-ligand interaction parameter, χ_G, includes enthalpic and entropic terms,

(S31) $\chi_G = \chi_H - T\chi_S$

Using the thermodynamic integration methodology,[7] we calculate χ_G, which provides excess free energy for mixing according to:

(S32) $\Delta G_{mix,ex} = \int_0^1 \langle\frac{\partial\mathcal{G}(\lambda)}{\partial\lambda}\rangle_\lambda\, d\lambda$

where λ, the integration parameter, represents the path for changing the Hamiltonian from a reference system (λ=0) to the system of interest (λ=1), and $\mathcal{G}$ is the system's free energy, calculated as the thermal average of $\mathcal{H}$. Note that here our reference point is chosen to be the ideally mixed state, for which the configurational mixing entropy is described by eq.(S26):

(S33) $\Delta G_{mix}^{id} = -RTln\frac{(L_A+L_B)!}{L_A!L_B!}$

where T is the experimental temperature (303K). Hence, the total ΔG_{mix} corresponding to the experimentally measured value ΔG_{mix}=0 is given by summing eqs. (S32) and (S33).

To simulate the NC surface, a square 11x11 grid (121 sites) with periodic boundary conditions was used to fit the total number of experimentally known surface sites (127, similar results were achieved for 12x12 grid). For a given number of L_A and L_B, the system was first initialized to a randomly mixed state (similar results were achieved when starting with a phase separated state). Then, the system was allowed to reach equilibrium by performing a large number of Monte Carlo steps. According to the Metropolis algorithm,[8] at each step, a trial swap between two ligands was suggested and the change in the system energy upon switching, ΔU, was calculated whereby each ligand interacts with its 4 neighbors: similar ligand pairs interact with 0 energy ($\chi_{G,AA}$=$\chi_{G,AB}$=0), and non-similar ligands interact with $\lambda\chi_G$. Swapping was allowed if it resulted in a decrease in the free energy, weighted by the Boltzmann probability,

(S34) $P_{switch} \propto \exp(\Delta\mathcal{G}\ /RT\ )$

After multiple steps, equilibrium was achieved, and the total energy of the system was calculated to give $\mathcal{G}(\lambda)$ for a specific λ. The procedure was repeated multiple times to give an averaged $\mathcal{G}(\lambda)$, which was later used for calculating ΔG_{mix} as described above.

The simulation was performed for several ratios of L_A and L_B, to give ΔG_{mix} over the full range of ligand molar fractions. As detailed in the main text, the interaction parameter χ_G which correspond to ΔG_{mix}=0 is 3.4RT, and this interaction parameter was used for simulating the ligand shell structure at equilibrium, using the Monte Carlo algorithm described here. The number of non-similar pairs (Figure 2 in the main text) and the average cluster area shown in Figure S13 were extracted from the simulated grid at equilibrium.

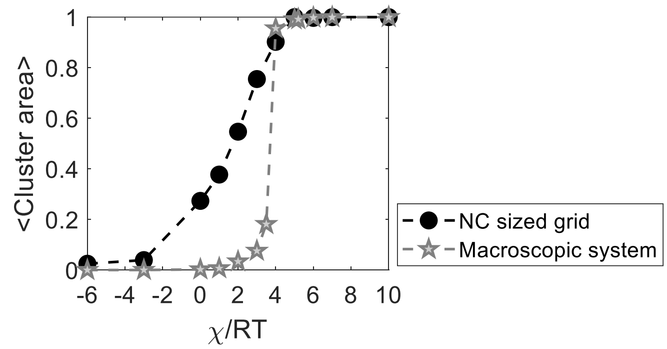

Figure S13. *Average cluster area as a function of the total interaction parameter, χ_G in simulation. Results are normalized to the maximal cluster size in the phase separation state, RT is the thermal energy, and the molar fraction of the surface ligands shown here is X=0.5. Results are presented for the NC sized grid (11x11), which exhibit a gradual change, and for the macroscopic system approaching the thermodynamic limit (111x111).Cluster area changes gradually for the NC, but increases abruptly around the reported phase transition point (~3.5RT)*[9,10] *in the large system.*

Fits for the enthalpy and the entropy (for each binary ligand set) are derived from the resulting χ_G. To resolve the entropic contribution to the free energy, we used the same thermodynamic integration methodology described above, but this time ΔG_{mix} was calculated for a temperature range of 297K to 309K (around the experimental temperature of 303K). The temperature dependence of χ_G was considered according to eq. (S31), allowing to determine χ_H and χ_S that best reproduce the experimental results. The enthalpy and the entropy for mixing were calculated from the temperature dependent simulation according to the van‘t Hoff relation:

(S35) $\Delta S_{mix} = -\frac{dG_{mix}}{dT};\ \Delta H_{mix} = \Delta G_{mix} + T\Delta S_{mix}$

`

As described in the main text, ΔS_{mix} is composed of configurational and non-configurational terms. ΔS_{mix}^{nc} is extracted by calculating the interactions (derived from χ_S) between all $L_A L_B$ pairs at equilibrium, for all simulated X_{C6SH}. Then, ΔS_{mix}^{conf}, is extracted by subtracting ΔS_{mix}^{nc} from the total ΔS_{mix}. Since ΔS_{mix}^{conf} represents the entropy of the ligand organization, which is directly derived from $\chi_{G,}$ systems with similar χ_G exhibit similar $\Delta S_{\mathrm{mix}}^{\mathrm{conf}}$.

For the C6SH:C6SH(F) mixture, a similar procedure was applied with the requirement of positive total ΔG_{mix} so as to match the ITC results.

For the C14SH:C18SH mixture, the simulation included in addition a linear dependence of χ_H and χ_S on ligand's molar fractions, as detailed in the main text. For completeness, we applied similar simulations also for the other investigated binary mixtures of C6SH:C10H, C6SH:C14SH, and C6SH:C18SH (Figure S14), which exhibit deviations from the fitting provided by constant χ_H and χ_S. However, we note that since the deviations from the original model were minor, the new model should be analyzed with caution so as to avoid over-interpretation. Comparing the fitting-extracted ω and η (Figure S14g and S14h, respectively), it is noticeable that the long ligands at each binary system induce loss of interaction energy and gain in entropy upon mixing. The parameters of C14SH, which represent the long ligand in the C6SH:C14SH system and the short ligand in the C14:SH:C18SH system, switch sign between both system, as the C14SH gain (loss) interactions and loss (gain) entropy upon mixing with the longer C18SH (shorter C6SH). The parameters for the C10SH and C6SH ligands are low and may vary due to using multiply fitting parameters to a system that could already be reasonably fitted with a single one (constant χ).

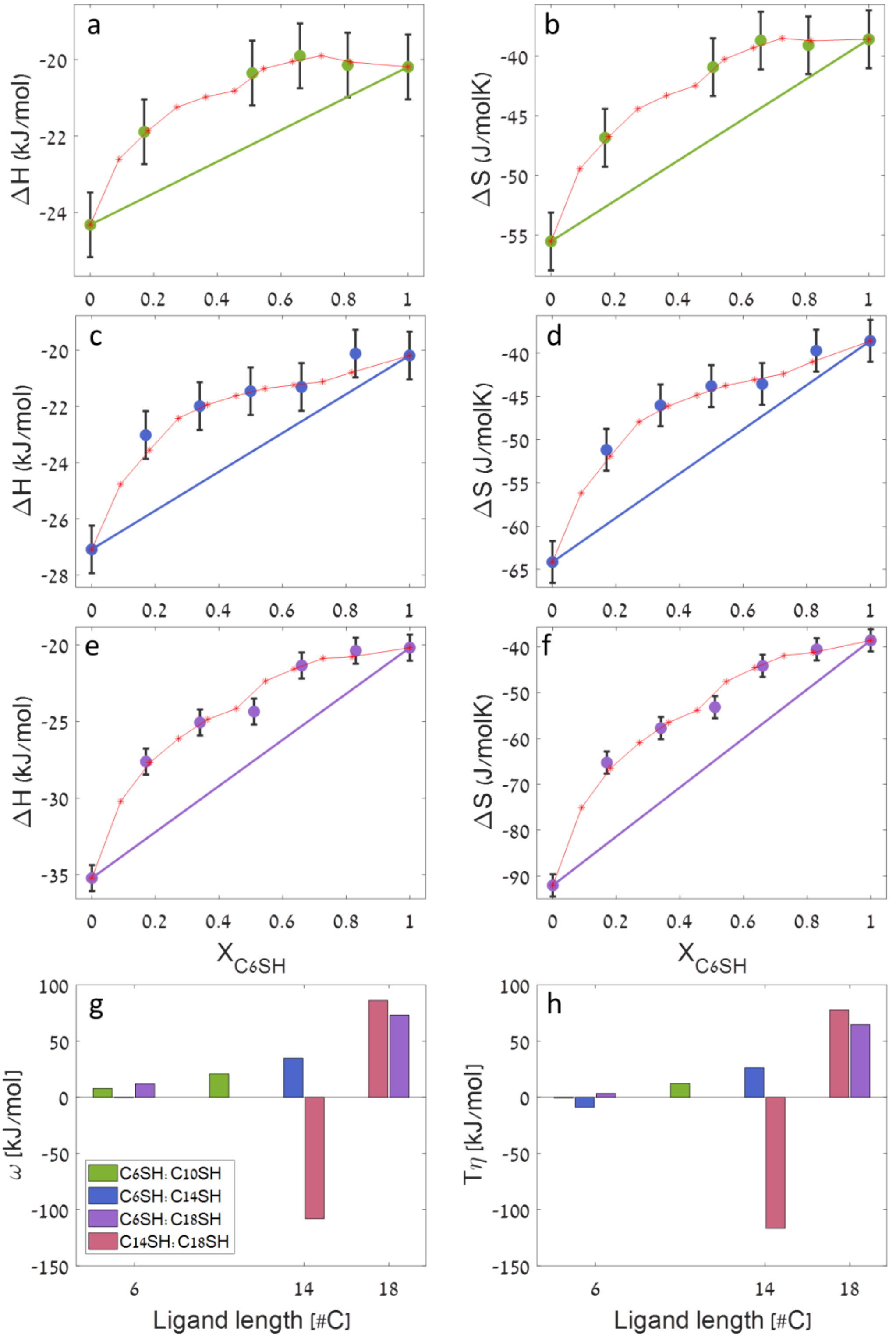

Figure S14. *ITC results (dots) and numerical fitting (red asterisks and lines) considering a composition dependent interaction parameters for (a-b) C6SH:C10SH, (c-d) C6SH:C14SH, and (e-f) C6SH:C18SH binary compositions. (g) Fitted enthalpic and (h) entropic interaction parameter coefficients for each ligand in the binary compositions.*

`

(1) Elimelech, O.; Aviv, O.; Oded, M.; Banin, U. A Tale of Tails: Thermodynamics of CdSe Nanocrystal Surface Ligand Exchange. *Nano Lett.* **2020**, *20* (9), 6396–6403.

(2) Elimelech, O.; Aviv, O.; Oded, M.; Peng, X.; Harries, D.; Banin, U. Entropy of Branching Out: Linear versus Branched Alkylthiols Ligands on CdSe Nanocrystals. *ACS Nano* **2022**, *16* (3), 4308–4321.

(3) Anderson, N. C.; Hendricks, M. P.; Choi, J. J.; Owen, J. S. Ligand Exchange and the Stoichiometry of Metal Chalcogenide Nanocrystals: Spectroscopic Observation of Facile Metal-Carboxylate Displacement and Binding. *J. Am. Chem. Soc.* **2013**, *135* (49), 18536–18548.

(4) Wiseman, T.; Williston, S.; Brandts, J. F.; Lin, L. N. Rapid Measurement of Binding Constants and Heats of Binding Using a New Titration Calorimeter. *Anal. Biochem.* **1989**, *179* (1), 131–137.

(5) Fritzinger, B.; Capek, R. K.; Lambert, K.; Martins, C.; Hens, Z. Utilizing Self-Exchange to Address the Binding of Carboxylic Acid Ligands to CdSe Quantum Dots. *J. Am. Chem. Soc.* **2010**, *132*, 10195–10201.

(6) Masel, R. I. *Principles of Adsorption and Reaction on Solid Surfaces*; John Wiley & Sons: New York, USA, 1996.

(7) Frenkel, D.; Smit, B. *Understanding Molecular Simulation: From Algorithms to Applications*; Academic Press: San Diego, 1996.

(8) Metropolis, N.; Rosenbluth, A. W.; Rosenbluth, M. N.; Teller, A. H.; Teller, E. Equation of State Calculations by Fast Computing Machines. *J. Chem. Phys.* **1953**, *21* (6), 1087–1092.

(9) Wannier, H. A. K. and G. H. Statistics of the Two-Dimensional Ferromagnet. Part I. *Phys. Rev.* **1941**, *60*, 252–262.

(10) Onsager, L. Crystal Statistics. I. A Two-Dimensional Model with an Order-Disorder Transition. *Phys. Rev.* **1944**, *65* (3–4), 117–149.
